# ﻿Hidden diversity of crust-like *Sebacinaceae* (*Sebacinales*, *Agaricomycetes*) in Asia

**DOI:** 10.3897/imafungus.17.168486

**Published:** 2026-01-14

**Authors:** Hannah Suh, Chang Wan Seo, Ki Hyeong Park, Shinnam Yoo, Dohye Kim, Yoonhee Cho, Young Woon Lim

**Affiliations:** 1 School of Biological Sciences and Institute of Biodiversity, Seoul National University, Seoul, Republic of Korea Seoul National University Seoul Republic of Korea; 2 Forest Entomology and Pathology Division, National Institute of Forest Science, Seoul, Republic of Korea National Institute of Forest Science Seoul Republic of Korea; 3 Department of Forest Biomaterials and Technology, Swedish University of Agricultural Sciences, Uppsala, Sweden Swedish University of Agricultural Sciences Uppsala Sweden

**Keywords:** Early-diverging species, ectomycorrhiza, *
Helvellosebacina
*, metabarcoding, new species, phylogeny, *
Sebacina
*, *
Tremelloscypha
*

## Abstract

Crust-like *Sebacinaceae*, comprising the genera *Helvellosebacina*, *Sebacina*, and *Tremelloscypha*, represent the only ectomycorrhizal lineage within the *Sebacinaceae* family. However, species delimitation within this group remains challenging because of their cryptic lifestyles, inconspicuous morphological traits, and limited taxonomic annotation. To address these limitations, we investigated crust-like *Sebacinaceae* in Asia by integrating two datasets: specimen-derived (barcoding) sequence data and root-associated metabarcoding data. A high diversity of crust-like *Sebacinaceae* species was uncovered, most of which did not match any previously described taxa. Multigene phylogenetic analyses (ITS, LSU, and *rpb*2) based on basidiomata identified eleven distinct species, of which six are proposed here as new to science. In parallel, metabarcoding data revealed additional crust-like *Sebacinaceae* species and confirmed their ectomycorrhizal association with *Pinus* and *Quercus* species. These findings advance our understanding of crust-like *Sebacinaceae* diversity and ecology in previously unexplored regions.

## ﻿Introduction

The family *Sebacinaceae* was proposed on the basis of key microscopic characteristics, notably similar basidial and hyphal features (Wells and Oberwinkler 1982). Within the early-diverging *Agaricomycetes* (Weiß and Oberwinkler 2001; Hibbett 2006), the order *Sebacinales* comprises two families, among which *Sebacinaceae* is unique as the only basidiomata-forming group (Oberwinkler et al. 2014). The basidiomata of *Sebacinaceae* are morphologically diverse, ranging from crust-like forms to erect structures, including cushion-, clavarioid-, and funnel-shaped forms (Oberwinkler et al. 2013). Microscopically, members of *Sebacinaceae* exhibit long sterigmata associated with longitudinally septate basidia and thick-walled hyphae with simple septa, resembling features found in *Auriculariales* and *Tremellales* (Wells et al. 2004; Oberwinkler et al. 2014; Millanes et al. 2015). The family *Sebacinaceae* comprises seven recognized genera: *Chaetospermum*, *Efibulobasidium*, *Globulisebacina*, *Helvellosebacina*, *Paulisebacina*, *Sebacina*, and *Tremelloscypha* (Oberwinkler et al. 2013; Weiß et al. 2016).

Ecologically, *Sebacinaceae* species perform diverse roles as saprotrophs, ectomycorrhizal symbionts, and potential multitrophic partners (Glen et al. 2002; Selosse et al. 2009). Among *Sebacinaceae*, ectomycorrhizal species are predominantly found in the genera *Helvellosebacina*, *Sebacina*, and *Tremelloscypha* (Oberwinkler et al. 2014). This ectomycorrhizal group is commonly found belowground as an ectomycorrhizal partner and plays critical ecological roles (Tedersoo and Smith 2013; Schön et al. 2022). Species in this group exhibit a simple crust-like morphology, which complicates their identification and leads to frequent misidentification, as has been reported for *Sebacina incrustans* (Riess et al. 2013; Oberwinkler et al. 2014). Because of this morphological simplicity, species in this family have often been overlooked. Based on these shared morphological characteristics, this ectomycorrhizal assemblage is hereafter referred to as the “crust-like *Sebacinaceae*.”

With the advancement of molecular techniques, DNA-based phylogenetic analysis has become a powerful tool for resolving taxonomic ambiguities and the diversity of *Sebacinaceae* (Weiß et al. 2004; Murphy et al. 2014; Sesli 2021). Although previous taxonomic studies on *Sebacinaceae* have utilized phylogenetic approaches, many relied on single genetic markers, lacked comprehensive taxon sampling, or included environmental sequences without taxonomic annotation (Moyersoen and Weiß 2014; Oberwinkler et al. 2014). To address these limitations, recent studies have emphasized the use of additional protein-coding markers, such as the RNA polymerase II subunit 2 gene (*rpb*2) (Riess et al. 2013; Tekpinar and Kalmer 2019), which provides improved species resolution and broad taxonomic coverage in *Sebacinaceae* (Riess et al. 2013).

Taxonomic studies of *Sebacinaceae* in Asia remain limited, hindering a comprehensive understanding of their true diversity (Kirschner et al. 2017; Dong et al. 2025). Currently, only a single *Sebacina* species (*S. incrustans*) has been reported from the Republic of Korea (Han et al. 2006; Lee et al. 2015). Additionally, two crust-like *Sebacinaceae* species (*Helvellosebacina filicata* and *Sebacina aciculicola*) have been newly reported from southwestern China (Dong et al. 2025), suggesting that crust-like *Sebacinaceae* taxa are present but underrepresented in Asian records. Meanwhile, recent metabarcoding studies have suggested that belowground fungal diversity is significantly underestimated (Sánchez-Ramírez et al. 2017; Alem et al. 2022), including the ectomycorrhizal crust-like *Sebacinaceae* group. Therefore, integrating basidiomata with metabarcoding data from environmental samples is crucial for a comprehensive understanding of diversity within *Sebacinaceae* (Schmidt et al. 2013; Abarenkov et al. 2024).

Given the need for extensive taxonomic research, we aimed to uncover the hidden diversity of crust-like *Sebacinaceae* in previously unexplored regions of Asia. This study integrated two types of data: barcoding data from basidiomata and metabarcoding data from the roots of *Pinus* and *Quercus* species, the dominant tree taxa in Korean forests. Based on a multigene phylogenetic analysis, including the internal transcribed spacer (ITS), nuclear large ribosomal subunit (LSU), and *rpb*2, we identified eleven distinct species from specimens collected in the Republic of Korea. Among them, six are proposed as new to science, for which we provide detailed morphological descriptions. Metabarcoding data further revealed additional crust-like *Sebacinaceae* species and confirmed their ectomycorrhizal association with *Pinus* and *Quercus* species. These findings significantly enhance our understanding of crust-like *Sebacinaceae* diversity and their ecological roles.

## ﻿Materials and methods

### ﻿Specimens studied

**Figure 1.**
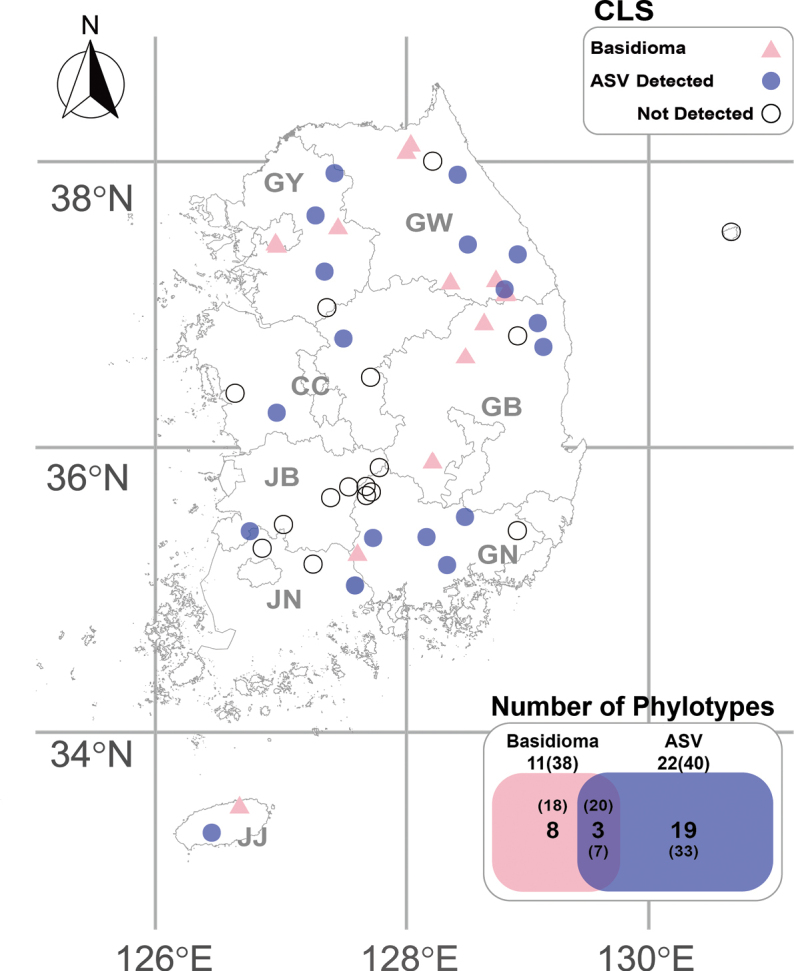
An overview of *Sebacinaceae* sampling sites and sample type comparison in Korea. Sampling sites include basidiomata (pink), root-associated metabarcoding data collection sites where crust-like *Sebacinaceae* were detected (blue), and sites where crust-like *Sebacinaceae* taxa were not detected (empty). The sample type comparison shown in the box indicates the number of crust-like *Sebacinaceae* taxa identified from basidiomata and root-associated metabarcoding data (ASV). Bold numbers indicate the number of each phylotype, and numbers in brackets represent the number of specimens or ASV counts.

A total of 38 *Sebacina*-like specimens were obtained from three fungaria in the Republic of Korea: the Seoul National University Fungus Collection (SFC), the National Institute of Biological Resources (NIBR), and the Korea University Culture Collection (KUC) (Table 1). All specimens were labeled as *S. incrustans*. Sampling was performed with permission from the Yang-gu Arboretum (Gangwon State, Republic of Korea). Collection information and photographs of the basidiomata taken in the field were obtained from each fungarium. Collection sites are shown in Fig. 1.

**Table 1.** List of *Sebacinaceae* vouchers and GenBank accession numbers used in this study.

Species	Voucher	GenBank accession number			Country	Reference
		ITS	LSU	*rpb2*		
*Chaetospermum artocarp*i	BCC18581	–	EF589735	–	Thailand	Rungjindamai et al. 2008
*C. camelliae*	CL-1	–	JQ794488	–	China	Kirschner et al. 2017
*C. chaetosporum*	CBS:154.59	–	NR_126146	NG_058876	Switzerland	Crous et al. 2014
	CBS:612.75	–	KJ710440	–	Pakistan	Crous et al. 2014
*Chaetospermum* sp.	RJB 12952	AF384860	AF384860	–	Unknown	Oberwinkler et al. 2014
*Ditangium altaicum*	LE 231836*	NR_163760	–	–	Russian Federation	Malysheva et al. 2019
*D. cerasi*	AFTOL-ID 1677	DQ520103	DQ520103	–	Unknown	Lutzoni et al. 2004
	TUB 020203	KF061265	KF061265	KF061300	Germany	Riess et al. 2014
	Wu 296	–	–	MN819826	China	Unpublished
*D. incarnatum*	LE 206311	MH836336	–	–	Russian Federation	Malysheva et al. 2019
	LE 303419	MH836337	–	–	Russian Federation	Malysheva et al. 2019
*Globulisebacina chenii*	R. Kirschner 3653	–	NG_070499	–	Taiwan	Kirschner et al. 2017
*G. rolleyi*	RJB 6889	–	AF291317	–	Canada	Wei and Oberwinkler 2001
	RJB 794	AY509550	AY509550	–	Unknown	Wells et al. 2004
*Helvellosebacina chrysallidodoma*	SFC20230908-01	**PV399946**	**PV399908**	**PV417267**	Republic of Korea	This study
	SFC20231006-06*	**PV399956**	**PV399918**	**PV417268**	Republic of Korea	This study
*H. concrescens*	RoKi_946	–	AY505545	–	China	Weiss et al. 2004
	TUB 019706	JQ665516	JQ665516	–	Gerany	Riess et al. 2013
*H. filicata*	Dai 20449*	PQ877259	PQ877258		China	Dong et al. 2025
*H. granulata*	PA-2020a	MT302587	MT302588	–	Turkey	Sesli 2021
*H. helvelloides*	TUB 019681	KJ546097	KJ546097	–	Austria	Oberwinkler et al. 2014
	TUB 019707	JQ665515	JQ665515	–	Germany	Riess et al. 2013
	TUB 020037	KF000465	KF000465	–	Germany	Oberwinkler et al. 2014
*H. koreana*	SFC20231005-04*	**PV399955**	**PV399917**	**PV417269**	Republic of Korea	This study
	SFC20231028-04	**PV399957**	**PV399919**	**PV417270**	Republic of Korea	This study
*Helvellosebacina* pt. 1	TUB 020021	KF000449	KF000449	–	Germany	Oberwinkler et al. 2014
	TUB 020028	KF000456	KF000456	–	Germany	Oberwinkler et al. 2014
*Helvellosebacina* pt. 2	TU 115570	UDB016423	UDB016423	UDB016423	United States of America	Hewitt et al. 2020
*Helvellosebacina* pt. 3	F1143128	DQ911617	DQ521412	–	United States of America	Lutzoni et al. 2004
*Helvellosebacina* pt. 4	F1143539	DQ521409	DQ521408	–	United States of America	Lutzoni et al. 2004
*Helvellosebacina* pt. 5	KNA_Sebacina_ASV28	**PV422651**	**PV422692**	–	Republic of Korea	This study
*Helvellosebacina* pt. 6	KNA_Sebacina_ASV31	**PV422654**	**PV422695**	–	Republic of Korea	This study
*Paulisebacina allantoidea*	RoKi 179	KF061266	AF291367	KF061301	Germany	Wei and Oberwinkler 2001
*Sebacina aciculicola*	Dai 25793*	PQ877260			China	Dong et al. 2025
	KNA_Sebacina_ASV01	**PV422624**	**PV422665**	–	Republic of Korea	This study
	KNA_Sebacina_ASV10	**PV422633**	**PV422674**	–	Republic of Korea	This study
	KNA_Sebacina_ASV16	**PV422639**	**PV422680**		Republic of Korea	This study
	KNA_Sebacina_ASV36	**PV422659**	**PV422700**		Republic of Korea	This study
	NIBRFG0000508545	**PV399923**	**PV399886**	**PV417255**	Republic of Korea	This study
	OTU 28_OM236634	OM236634	–	–	Republic of Korea	Yoo et al. 2022
	SeqID43	OR482699	–	–	China	Dong et al. 2025
	SFC20230802-01	**PV399935**	**PV399897**	**PV417257**	Republic of Korea	This study
	SFC20230817-46	**PV399943**	**PV399905**	–	Republic of Korea	This study
	SFC20230908-06	**PV399951**	**PV399913**	**PV417256**	Republic of Korea	This study
*S. aureomagnifica*	GT-2015(JPB60535)	LN868956	–	–	Brazil	Wartchow et al. 2015
*S. caducifoliicola*	SFC20230704-04	**PV399930**	**PV399892**	**PV417273**	Republic of Korea	This study
	SFC20230704-05	**PV399931**	**PV399893**	**PV417274**	Republic of Korea	This study
	SFC20230817-41*	**PV399940**	**PV399902**	**PV417275**	Republic of Korea	This study
	SFC20230817-45	**PV399942**	**PV399904**	**PV417276**	Republic of Korea	This study
*S. crystallina*	NIBRFG0000504700	**PV399921**	**PV399884**	–	Russian Federation	This study
	SFC20190919-11*	**PV399927**	**PV399890**	**PV417271**	Republic of Korea	This study
	SFC20230729-09	**PV399934**	**PV399896**	**PV417272**	Republic of Korea	This study
*S. cystidiata*	TUB 020022	KF000450	KF000450	–	Germany	Oberwinkler et al. 2014
	TUB 020024*	NR_154609	KF000452	–	Germany	Oberwinkler et al. 2014
	TUB 020025	KF000453	KF000453	–	Germany	Oberwinkler et al. 2014
*S. dimitica*	KNA_Sebacina_ASV04	**PV422627**	**PV422668**	–	Republic of Korea	This study
	KNA_Sebacina_ASV05	**PV422628**	**PV422669**	–	Republic of Korea	This study
	KNA_Sebacina_ASV06	**PV422629**	**PV422670**	–	Republic of Korea	This study
	KNA_Sebacina_ASV21	**PV422644**	**PV422685**	–	Republic of Korea	This study
	KNA_Sebacina_ASV25	**PV422648**	**PV422689**	–	Republic of Korea	This study
	KNA_Sebacina_ASV34	**PV422657**	**PV422698**	–	Republic of Korea	This study
	MW 525	–	AF291364	–	Germany	Wei and Oberwinkler 2001
	OTU 86_OM236585	OM236585		–	Republic of Korea	Yoo et al. 2022
	TAA169135	KF061274	KF061274	–	Estonia	Oberwinkler et al. 2014
	TUB 019988	KF061272	KF061272	KF061302	Austria	Riess et al. 2014
*S. epigaea*	KNA_Sebacina_ASV15	**PV422638**	**PV422679**	–	Republic of Korea	This study
	KNA_Sebacina_ASV17	**PV422640**	**PV422681**	–	Republic of Korea	This study
	KNA_Sebacina_ASV26	**PV422649**	**PV422690**	–	Republic of Korea	This study
	KNA_Sebacina_ASV30	**PV422653**	**PV422694**	–	Republic of Korea	This study
	KNA_Sebacina_ASV39	**PV422662**	**PV422703**	–	Republic of Korea	This study
	KNA_Sebacina_ASV40	**PV422663**	**PV422704**	–	Republic of Korea	This study
	TUB 019670	JQ665490	JQ665490	JQ665589	Germany	Riess et al. 2013
	TUB 019671	JQ665486	JQ665486	JQ665585	Germany	Riess et al. 2013
*S. flagelliformis*	TUB 019669	JQ665497	JQ665497	–	Germany	Riess et al. 2013
	TUB 020035	KF000463	KF000463	–	Germany	Oberwinkler et al. 2014
	TUB 020036*	NR_138387	KF000464	–	Germany	Oberwinkler et al. 2014
*S. guayanensis*	BM03M3	JQ063056	KF773775	–	Venezuela	Moyersoen and Weiss 2014
*S. incarnata*	SFC20200821-21*	**PV399928**	**PV399891**	–	Republic of Korea	This study
	SFC20200821-26	**PV399929**	–	–	Republic of Korea	This study
*S. incrustans*	LKH Obj-97-2	–	FJ644513	FJ623653	United States of America	Kottke et al. 2010
	MW 524	AF490395	AF291365	–	Germany	Wei and Oberwinkler 2001
	TUB 019604	JQ665534	JQ665534	JQ665641	Germany	Riess et al. 2013
	TUB 019605	JQ665532	JQ665532	JQ665640	Germany	Riess et al. 2013
*S. ocreata*	KNA_Sebacina_ASV07	**PV422630**	**PV422671**	–	Republic of Korea	This study
	KNA_Sebacina_ASV09	**PV422632**	**PV422673**	–	Republic of Korea	This study
	MAS1	AF440664	AF440664	–	France	Selosse et al. 2002
	MCA 2069	–	AY393696	–	Guyana	Henkel et al. 2004
	SFC20130917-H16	**PV399924**	**PV399887**	**PV417252**	Republic of Korea	This study
	SFC20150701-21	**PV399925**	**PV399888**	**PV417251**	Republic of Korea	This study
	SFC20230722-33	**PV399933**	**PV399895**	–	Republic of Korea	This study
	SFC20230816-52	**PV399936**	**PV399898**	–	Republic of Korea	This study
	SFC20230816-56	**PV399938**	**PV399900**	–	Republic of Korea	This study
	SFC20230817-39	**PV399939**	**PV399901**	**PV417253**	Republic of Korea	This study
	SFC20230908-02	**PV399947**	**PV399909**	**PV417254**	Republic of Korea	This study
	TUB 019637	JQ665548	JQ665548	JQ665619	Germany	Riess et al. 2013
	TUB 020011	KF000440	KF000440	–	Germany	Oberwinkler et al. 2014
*S. orientalis*	KNA_Sebacina_ASV13	**PV422636**	**PV422677**	–	Republic of Korea	This study
	SFC20230816-55	**PV399937**	**PV399899**	–	Republic of Korea	This study
	SFC20230817-43	**PV399941**	**PV399903**	**PV417258**	Republic of Korea	This study
	SFC20230907-02	**PV399944**	**PV399906**	–	Republic of Korea	This study
	SFC20230907-03	**PV399945**	**PV399907**	**PV417259**	Republic of Korea	This study
	SFC20230908-03	**PV399948**	**PV399910**	–	Republic of Korea	This study
	SFC20230908-04	**PV399949**	**PV399911**	–	Republic of Korea	This study
	SFC20230908-05	**PV399950**	**PV399912**	**PV417260**	Republic of Korea	This study
	SFC20230908-13*	**PV399952**	**PV399914**	**PV417262**	Republic of Korea	This study
	SFC20230908-19	**PV399953**	**PV399915**	**PV417261**	Republic of Korea	This study
*S. pseudocandida*	AFTOL-ID 699	DQ411526	AY745701	DQ408132	United States of America	Geml et al. 2014
	TUB 020330	KF061277	KF061277	KF061304	United States of America	Riess et al. 2014
	TUB 020331	KF061278	KF061278	KF061305	United States of America	Riess et al. 2014
*S. schweinitzii*	TUB 020209	–	KF061276	–	United States of America	Riess et al. 2014
	TUB 019645	JQ665559	JQ665559	JQ665656	Germany	Riess et al. 2013
	TUB 019646	JQ665563	JQ665563	JQ665655	Germany	Riess et al. 2013
*S. tomentosa*	PD10	KF773779	KF773779	–	Venezuela	Moyersoen and Weiss 2014
*Sebacina* pt. 01	SFC20230704-09	**PV399932**	**PV399894**	–	Republic of Korea	This study
*Sebacina* pt. 02	KNA_Sebacina_ASV14	**PV422637**	**PV422678**	–	Republic of Korea	This study
*Sebacina* pt. 03	KNA_Sebacina_ASV11	**PV422634**	**PV422675**	–	Republic of Korea	This study
*Sebacina* pt. 04	KNA_Sebacina_ASV32	**PV422655**	**PV422696**	–	Republic of Korea	This study
*Sebacina* pt. 05	KNA_Sebacina_ASV03	**PV422626**	**PV422667**	–	Republic of Korea	This study
	KNA_Sebacina_ASV08	**PV422631**	**PV422672**	–	Republic of Korea	This study
	KNA_Sebacina_ASV22	**PV422645**	**PV422686**	–	Republic of Korea	This study
	KNA_Sebacina_ASV24	**PV422647**	**PV422688**	–	Republic of Korea	This study
*Sebacina* pt. 06	KNA_Sebacina_ASV37	**PV422660**	**PV422701**	–	Republic of Korea	This study
*Sebacina* pt. 07	KNA_Sebacina_ASV19	**PV422642**	**PV422683**	–	Republic of Korea	This study
*Sebacina* pt. 08	KNA_Sebacina_ASV35	**PV422658**	**PV422699**	–	Republic of Korea	This study
*Sebacina* pt. 09	KNA_Sebacina_ASV27	**PV422650**	**PV422691**	–	Republic of Korea	This study
*Sebacina* pt. 10	KNA_Sebacina_ASV29	**PV422652**	**PV422693**	–	Republic of Korea	This study
	OTU 17_OM236623	OM236623	–	–	Republic of Korea	Yoo et al. 2022
	OTU 89_OM236586	OM236586	–	–	Republic of Korea	Yoo et al. 2022
	OTU 136_OM236593	OM236593	–	–	Republic of Korea	Yoo et al. 2022
*Sebacina* pt. 11	OTU 51_OM236655	OM236655	–	–	Republic of Korea	Yoo et al. 2022
*Sebacina* pt. 12	SFC20231005-01	**PV399954**	**PV399916**	**PV417266**	Republic of Korea	This study
*Sebacina* pt. 13	TUB 020023	KF000451	KF000451	–	Germany	Oberwinkler et al. 2014
	Y104	–	AY505560	–	Austria	Weiss et al. 2004
*Sebacina* pt. 14	TUB 020029	KF000457	KF000457	–	Germany	Oberwinkler et al. 2014
*Sebacina* pt. 15	MW 526	AF490393	AF291363	–	Germany	Matheny et al. 2007
	TUB 020007	KF000436	KF000436	–	Germany	Oberwinkler et al. 2014
*Sebacina* pt. 16	KNA_Sebacina_ASV20	**PV422643**	**PV422684**	–	Republic of Korea	This study
*Sebacina* pt. 17	TUB 019999	KF000428	KF000428	–	Austria	Oberwinkler et al. 2014
	TUB 020017	KF000445	KF000445	–	Germany	Oberwinkler et al. 2014
*Sebacina* pt. 18	KNA_Sebacina_ASV23	**PV422646**	**PV422687**	–	Republic of Korea	This study
	TUB 020005	KF000434	KF000434	–	Austria	Oberwinkler et al. 2014
	WT21_DQ273443	–	DQ273443	–	United States of America	Bergemann and Garbelotto 2006
*Sebacina* pt. 19	FO41103	–	AY505544	–	United States of America	Weiss et al. 2004
*Sebacina* pt. 20	KNA_Sebacina_ASV02	**PV422625**	**PV422666**	–	Republic of Korea	This study
*Sebacina* pt. 21	KNA_Sebacina_ASV18	**PV422641**	**PV422682**	–	Republic of Korea	This study
*Sebacina* pt. 22	KUC20230817-19	**PV399920**	**PV399883**	**PV417263**	Republic of Korea	This study
	NIBRFG0000505680	**PV399922**	**PV399885**	**PV417264**	Republic of Korea	This study
	SFC20190820-11	**PV399926**	**PV399889**	**PV417265**	Republic of Korea	This study
*Sebacina* pt. 23	KNA_Sebacina_ASV38	**PV422661**	**PV422702**	–	Republic of Korea	This study
*Sebacina* pt. 24	AFTOL-ID 1876	DQ520095	DQ520095	–	Germany	Geml et al. 2014
*Sebacina* pt. 25	KNA_Sebacina_ASV12	**PV422635**	**PV422676**	–	Republic of Korea	This study
	KNA_Sebacina_ASV33	**PV422656**	**PV422697**	–	Republic of Korea	This study
*Serendipita petricolae*	JDEADA2.4	OL679100	OM327581	–	Australia	Crous et al. 2022
*Serendipita restingae*	MN595219	MN595219	MN595219	–	Brazil	Fritsche et al. 2021
*Serendipita vermifera*	MAFF 305842	KF061298	KF061298	KF061261	Australia	Riess et al. 2014
*Tremelloscypha dichroa*	Ryvarden 45376	KF061283	KF061283	KF061308	Puerto Rico	Oberwinkler et al. 2014
*T. gelatinosa*	GG 23605	AF490394	AF291376	–	Germany	Selosse et al. 2002
	LM4353	KF061279	KF061279	KF061306	Mexico	Riess et al. 2014
*Tremelloscypha* sp. “*pileata*”	KM133631	KF307630	–	–	Unknown	Murphy et al. 2014

Accession numbers of all sequences generated in this study are bolded in black. Type species are denoted by asterisks (*) on their vouchers.

### ﻿DNA extraction, PCR amplification, and sequencing

Genomic DNA was extracted from dried specimens (1 cm × 1 cm tissue sections) using the CTAB protocol (Rogers and Bendich 1994) and the AccuPrep® Genomic DNA Extraction Kit (Bioneer, Daejeon, Republic of Korea). Polymerase chain reaction (PCR) was performed for the ITS, LSU, and *rpb*2 regions. The ITS region was amplified using primers ITS5/ITS4-Seb (White et al. 1990; Tedersoo et al. 2011). Primers LR0R/LR5-Seb (Vilgalys and Hester 1990; Tedersoo et al. 2008) were used to amplify the LSU region. For the protein-coding gene, the *rpb*2 region was amplified using primers RPB2-Seb500f/RPB2-Seb1200rev (Tedersoo et al. 2014). PCR was performed following previously described protocols (Suh et al. 2024).

PCR products were verified by gel electrophoresis using a 1% agarose gel with EcoDye DNA staining solution (SolGent Co., Daejeon, Republic of Korea). The products were purified using the ExoSAP-IT Express PCR Product Cleanup Kit (Thermo Fisher Scientific, Waltham, MA, USA), following the manufacturer’s instructions. Sequencing was performed by Macrogen (Seoul, Republic of Korea) using an ABI 3730xl DNA analyzer (Life Technologies, Carlsbad, CA, USA). All sequences were proofread and edited using MEGA version 11 (Tamura et al. 2021) and FinChTV v.1.4. All newly generated sequences were deposited in GenBank, and their accession numbers are provided in Table 1.

### ﻿Root DNA metabarcoding

Separate from the *Sebacina* basidiomata collection, root samples were independently collected alongside specimen collection at 34 sites across the Korean Peninsula between 2019 and 2021, with permission from the Korean Government. At each site, five individuals of conifer (*Pinus densiflora*) and five individuals of oak (either *Quercus mongolica* or *Q. salicina*) were randomly selected, and lateral roots were excised from two opposite directions. Soil and debris adhering to the lateral roots were removed by gently shaking the samples in distilled water. All fine roots with fresh, healthy-appearing tips were separated using sterilized scissors. The separated fine roots were sterilized by submerging them in 3% hydrogen peroxide for 1 min, followed by three rinses with sterile distilled water to remove residual contaminants. Sterilized roots from each tree were pooled as a single sample and air-dried overnight on sterilized filter paper in a clean bench. Dried root tissues were finely ground in an autoclaved mortar using liquid nitrogen.

A total of 340 root samples were used for DNA metabarcoding. DNA extraction and amplicon library preparation targeting the ITS and LSU regions were conducted as described by Park et al. (2021). SMRTbell library preparation, sequencing, HiFi read generation, and demultiplexing were carried out at Macrogen (Seoul, Republic of Korea) using the PacBio Sequel platform. Demultiplexed amplicon reads were processed using QIIME2 (Bolyen et al. 2019). Adapters were filtered using Cutadapt (Martin 2011). Raw reads were denoised into amplicon sequence variants (ASVs) using DADA2 (Callahan et al. 2016). ASVs lacking ITS or LSU regions were filtered and trimmed using ITSx (Bengtsson-Palme et al. 2013). Taxonomic classification of ASVs was performed using a q2-feature-classifier against the UNITE database version 8 (Tedersoo et al. 2018), and ASVs assigned to crust-like *Sebacinaceae* were selected (Table 2). The crust-like *Sebacinaceae*ASVs were divided into ITS and LSU regions based on the ITSx results and used for subsequent phylogenetic analyses.

**Table 2.** Amplicon sequence variants (ASV) table.

ASV	Accession		Host†	Location‡	Taxonomy assigned
	ITS	LSU			
ASV01	PV422624	PV422665	Pd	GY	*Sebacina aciculicola*
ASV02	PV422625	PV422666	Pd/Qs	CC(Qs), GY(Pd)	*Sebacina* pt. 20
ASV03	PV422626	PV422667	Pd	JJ/JN	*Sebacina* pt. 5
ASV04	PV422627	PV422668	Pd	GY	*Sebacina dimitica*
ASV05	PV422628	PV422669	Pd	GN	*Sebacina dimitica*
ASV06	PV422629	PV422670	Pd/Qs	GB(Qs), GY(Pd)	*Sebacina dimitica*
ASV07	PV422630	PV422671	Pd	GN	*Sebacina ocreata*
ASV08	PV422631	PV422672	Pd	GW	*Sebacina* pt. 5
ASV09	PV422632	PV422673	Pd	JN	*Sebacina ocreata*
ASV10	PV422633	PV422674	Pd	CC, GW, JN	*Sebacina aciculicola*
ASV11	PV422634	PV422675	Qs	GY	*Sebacina* pt. 3
ASV12	PV422635	PV422676	Pd	GN,	*Sebacina* pt. 25
ASV13	PV422626	PV422677	Pd	JN	*Sebacina orientalis*
ASV14	PV422637	PV422678	Qs	GY	*Sebacina* pt. 2
ASV15	PV422638	PV422679	Pd	GN	*Sebacina epigaea*
ASV16	PV422639	PV422680	Qs	CC, GY	*Sebacina aciculicola*
ASV17	PV422640	PV422681	Qs	GN	*Sebacina epigaea*
ASV18	PV422641	PV422682	Pd	JJ	*Sebacina* pt. 21
ASV19	PV422642	PV422683	Pd	GW	*Sebacina* pt. 7
ASV20	PV422643	PV422684	Qs	GN	*Sebacina* pt. 16
ASV21	PV422644	PV422685	Pd	JJ	*Sebacina dimitica*
ASV22	PV422645	PV422686	Qs	GW	*Sebacina* pt. 5
ASV23	PV422646	PV422687	Qs	CC	*Sebacina* pt. 18
ASV24	PV422647	PV422688	Pd	GN	*Sebacina* pt. 5
ASV25	PV422648	PV422689	Pd/Qs	GB(Qs), GN(Pd)	*Sebacina dimitica*
ASV26	PV422649	PV422690	Qs	GW	*Sebacina epigaea*
ASV27	PV422650	PV422691	Qs	GW	*Sebacina* pt. 9
ASV28	PV422651	PV422692	Qs	GW	*Helvellosebacina* pt. 5
ASV29	PV422652	PV422678	Qs	UL	*Sebacina* pt. 10
ASV30	PV422653	PV422693	Qs	UL	*Sebacina epigaea*
ASV31	PV422654	PV422694	Qs	UL	*Helvellosebacina* pt. 6
ASV32	PV422655	PV422695	Qs	GN	*Sebacina* pt. 4
ASV33	PV422656	PV422696	Pd	GW(Pd)	*Sebacina* pt. 25
ASV34	PV422657	PV422697	Qs	GY	*Sebacina dimitica*
ASV35	PV422658	PV422698	Qs	CC	Sebacina pt. 8
ASV36	PV422659	PV422699	Qs	GY	*Sebacina aciculicola*
ASV37	PV422660	PV422700	Pd	CC	*Sebacina* pt. 6
ASV38	PV422661	PV422701	Qs	CC	*Sebacina* pt. 23
ASV39	PV422662	PV422702	Qs	JN	*Sebacina epigaea*
ASV40	PV422663	PV422703	Qs	GY	*Sebacina epigaea*

†Host: Pd–*Pinus densiflora*, Qs–*Quercus* spp. ‡Location: CC (Chungcheongbuk-do and Chungcheongnam-do), GB (Gyeongsangbuk-do), GN (Gyeongsangnam-do), GW (Gangwon State), GY (Gyeonggi-do), JB (Jeonbuk-State), JJ (Jeju), JN (Jeollanam-do), and UL (Ulleung-gun) §Q2 refers to the molecular identification based on Q2 classifier information. |Confidence indicates the reliability of a specific taxonomic rank based on q2 classifier information.

### ﻿Phylogenetic analysis

To increase the resolution and reliability of the phylogenetic tree, a multigene phylogeny based on ITS, LSU, and *rpb*2 sequences was assessed. Reference sequences of *Sebacinaceae* were retrieved from published taxonomic studies using GenMine (Seo et al. 2022). A total of 26 reliable reference datasets were obtained from previous studies, including basidiomata sequences reported in 20 publications and root tip sequences from Selosse et al. (2002), Moyersoen and Weiß (2014), Hewitt et al. (2020), and Yoo et al. (2022). Three species from *Serendipitaceae*—*Serendipita restingae*, *S. petricolae*, and *S. vermifera*—were selected as outgroups (Fritsche et al. 2021; Crous et al. 2022). To verify the phylogenetic position of each new species, phylogenetic analyses were performed, including all available ITS sequences from GenBank with BLAST percent identity equal to or greater than that of each new species’ closest sister taxon. Sequence processing, multiple sequence alignment, trimming, concatenation, model selection, and maximum likelihood (ML) tree inference were conducted using FunVIP v0.4.1 with the “accurate” preset (Seo et al. 2025). All results were manually validated to ensure accuracy.

### ﻿Morphology

For macroscopic features, the basidiomata surface and margin were described based on field photographs and notes. Microscopic examination of the dried specimens was performed by mounting the tissue in 5% KOH on a glass slide. The basidia, basidiospores, and hyphae were observed using a Nikon Eclipse 80i microscope (Nikon, Japan), and their sizes were measured using ImageJ software (Collins 2007). Colors were described based on the *Methuen Handbook of Colour* (Kornerup and Wanscher 1978). The following abbreviations are used: IKI = Melzer’s reagent, IKI− = neither amyloid nor dextrinoid, CB = Cotton Blue, CB− = acyanophilous, L = mean spore length, W = mean spore width, Q = L/W ratio of spores, and n = number of spores measured from the given number of specimens.

## ﻿Results

### ﻿Molecular phylogeny

A total of 38 crust-like *Sebacinaceae* specimens, newly collected and obtained from herbaria, were sequenced for the ITS and LSU regions. Based on sequence similarity and phylogenetic clustering, these specimens were delimited as 11 distinct taxa. To enhance phylogenetic resolution among these taxa, 26 *rpb*2 sequences were additionally sequenced from representative specimens of each taxon.

Metabarcoding analysis was conducted using 340 root samples from 34 sites across Korea, including all provinces nationwide (Fig. 1). The root-associated ASVs of *P. densiflora*, *Q. mongolica*, and *Q. salicina* were obtained separately for each host species. As a result, 2,243,343 HiFi reads were generated via PacBio sequencing, with an average of 6,598 reads per sample. After DADA2 denoising and singleton filtering, the sequence data yielded 3,439 ASVs. Using the q2-feature-classifier, 40 ASVs were identified as crust-like *Sebacinaceae* and verified through phylogenetic analysis together with barcoding data. Each ASV was split into separate ITS and LSU sequences, resulting in 40 ITS and 40 LSU sequences for phylogenetic analysis. Of the 40 crust-like SebacinaceaeASVs detected across 18 sites, 33 ASVs (representing 19 phylotypes) were detected exclusively through metabarcoding, and only seven ASVs (corresponding to three phylotypes) matched species identified from basidiomata (Fig. 1).

**Figure 2.**
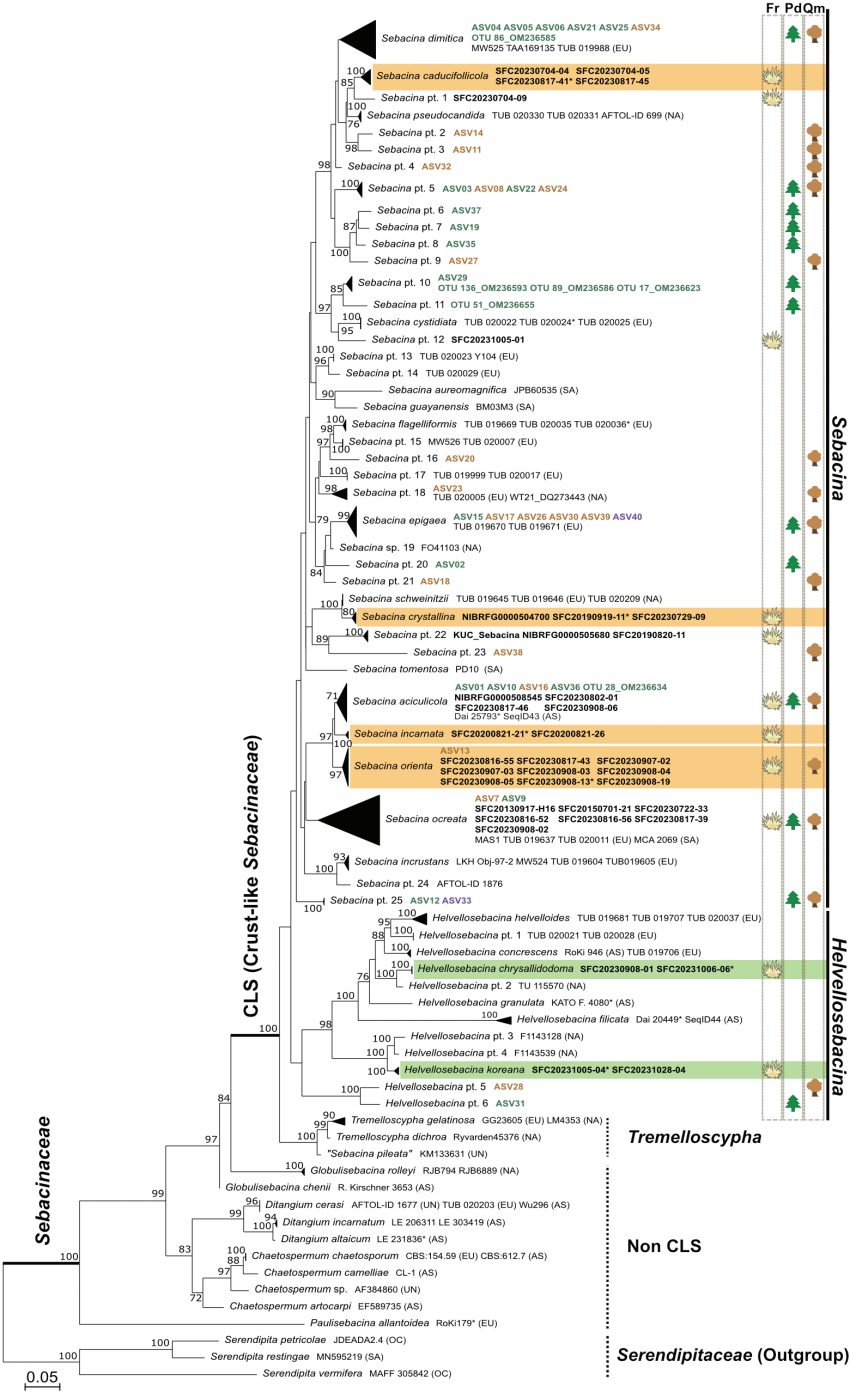
Phylogenetic tree inferred from maximum likelihood (ML) analysis based on ITS, LSU, and *rpb*2 sequences for the family *Sebacinaceae* and outgroups. ML bootstrap support values above 70 are shown at the nodes. Specimen voucher or strain numbers are given after each species name. The continental origin of each specimen is indicated in brackets: AS (Asia), EU (Europe), NA (North America), OC (Oceania), SA (South America), and UN (unknown). *Sebacina* pt. refers to phylotype data consisting of unknown species based on basidiomata or metabarcoding data. New species are highlighted in orange for *Sebacina* and green for *Helvellosebacina*. Basidiomata from this study are in bold, with a yellow crust-like symbol on the right. Metabarcoding data from Korea are bolded in green for *Pinus densiflora*, brown for *Quercus* spp., and purple for samples associated with both hosts. Green and brown host tree symbols on the right indicate *Pinus densiflora* and *Quercus* spp., respectively. Specimens marked with an asterisk (*) indicate holotypes.

In total, the dataset used for phylogenetic inference comprised 141 ITS, 143 LSU, and 46 *rpb*2 sequences (Table 1). The concatenated alignment was 2,800 nucleotides in length, including gaps, with ITS = 830 bases, LSU = 915 bases, and *rpb*2 = 1,055 bases. The maximum likelihood phylogeny based on the concatenated dataset (ITS + LSU + *rpb*2) clearly distinguished crust-like *Sebacinaceae* from non-crust-like *Sebacinaceae* lineages. However, it did not strongly support separation between *Sebacina* and *Helvellosebacina* within *Sebacinaceae*. Several non-crust-like *Sebacinaceae* genera—including *Chaetospermum*, *Ditangium*, *Globulisebacina*, and *Paulisebacina*—were placed in basal positions relative to the crust-like *Sebacinaceae* clade (Fig. 2). Independently formed clades were treated as phylotypes, reflecting well-supported monophyletic lineages. In total, 41 phylotypes were identified within *Sebacina*, 12 within *Helvellosebacina*, and three within *Tremelloscypha*. No *Tremelloscypha* species were detected among Korean samples.

Most phylotypes newly included in this study did not match previously described species. Among those forming clades comprising two or more basidiomata, six were proposed as new species—four within *Sebacina* and two within *Helvellosebacina*. The ITS phylogenetic trees based on all sequences with ≥ 94% similarity, the threshold determined by the closest sister taxon, are presented in Suppl. material 1: figs S1–S4. Phylotypes represented by a single specimen, lacking distinctive morphological features even when multiple specimens were available (e.g., *Sebacina* pt. 22), or detected solely through ASVs were retained as unnamed phylotypes. *S. aciculicola*, *S. ocreata*, and *S. orientalis* were detected in both basidiomata and metabarcoding datasets, whereas the remaining species were restricted to either source. Species identified exclusively through basidiomata include *H. chrysallidodoma*, *H. koreana*, *S. caducifoliicola*, *S. crystallina*, and *S. incarnata*. In contrast, some species, such as *S. dimitica* and *S. epigaea*, previously documented based on basidiomata material in Europe, were detected only through metabarcoding data.

Ecologically, based on analyses of 40 ASVs (22 phylotypes) and six reference sequences (four phylotypes)—the latter obtained from Korean *Pinus densiflora* seedlings (Yoo et al. 2021)—13 phylotypes were detected in the roots of *Pinus* hosts and 16 in *Quercus* hosts. Six phylotypes (*Sebacina aciculicola*, *S. dimitica*, *S. epigaea*, *S. ocreata*, *Sebacina* pt. 5, and *Sebacina* pt. 25) were found in both *Pinus* and *Quercus* roots (Fig. 2).

### ﻿Taxonomy

A total of 38 basidiomata specimens were ultimately identified as 11 species, comprising nine *Sebacina* species and two *Helvellosebacina* species. Except for *S. aciculicola* and *S. ocreata*, nine taxa were considered new species candidates with no prior global records. However, *Sebacina* pt. 1 and *Sebacina* pt. 12 were not proposed as new species because each was represented by a single specimen, whereas *Sebacina* pt. 22 was excluded because it lacked reliable diagnostic morphological features. Therefore, we propose six new species: *Helvellosebacina chrysallidodoma*, *Helvellosebacina koreana*, *Sebacina caducifoliicola*, *Sebacina crystallina*, *Sebacina incarnata*, and *Sebacina orientalis*. Six new species and two newly recorded species of crust-like *Sebacinaceae* from Korea are described below based on their morphological characteristics.

#### ﻿*Helvellosebacina* Oberw., Garnica & K. Riess, Mycol. Progr. 13(3): 467. 2014.

##### ﻿ Helvellosebacina chrysallidodoma

Taxon classificationAnimaliaSebacinalesSebacinaceae

H. Suh, D. Kim & Y.W. Lim sp. nov.

819854AB-7EB2-5BEC-88A2-C38C0342B443

858530

Fig. 3

###### Diagnosis.

Basidiomata grow on small shrubs, forming a pupal shelter-like structure covering the entire trunk.

**Figure 3.**
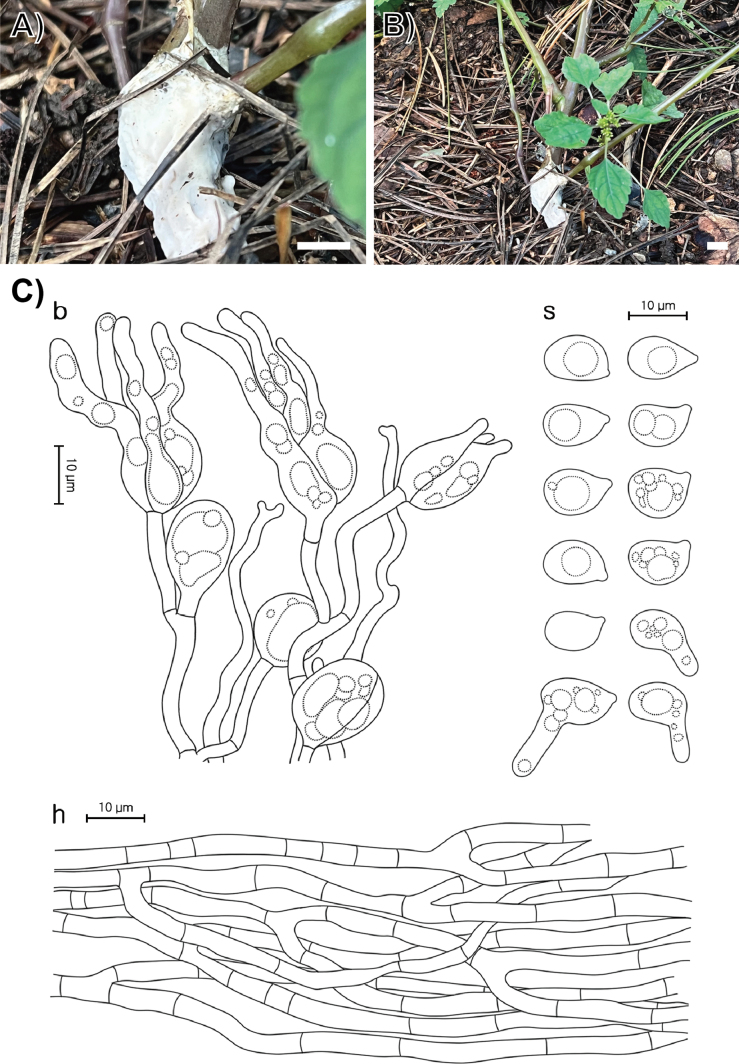
Morphological characters of *Helvellosebacina chrysallidodoma*. **A** Basidiomata (SFC20231006-06, holotype); **B** basidiomata (SFC20230908-01); **C** microscopic features, where ‘b’ refers to basidia, ‘s’ to basidiospores, and ‘h’ to hyphae. Scale bar: 1 cm (**A, B**).

###### Holotype.

REPUBLIC OF KOREA, Gyeonggi-do, Gapyeong-gun, Mt. Yumyeong Natural Recreation Forest, on a shrub stem, 06 October 2023, SFC20231006-06 (dried specimen).

###### Etymology.

From *chrysallidis*–pupa and *doma*–house in Latin, referring to the resemblance of the basidiomata that surround a trunk like a pupal shelter.

###### Description.

Basidiomata fully resupinate, attached firmly to the shrub stems, forming thick wax-like patches covering the whole substrate, spanning several centimeters. Surface smooth, dull, white (1A1), consistency wax-like, cartilaginous. Margin irregularly thinning out, distinctly bounded, concolorous with hymenophore surface. Basal hyphal system monomitic, subicular hyphae average 0.5 μm thick, septate with frequent branching, hyaline, without clamp connections, 2.8–4.2 μm thick. Basidiospores elliptical or allantoid, hyaline, smooth, IKI−, CB−, (8.0–)8.6–13.6(–14.7) × (6.1–)6.6–9.3(–10.3) μm with granular contents (L = 10.8 μm, W = 7.7 μm, Q = 1.4, n = 35/2). Dikaryophyses simple-structured, sparingly ramified, derived from the same hyphae with basidia. Probasidia elliptical to subglobose or pyriform, mature basidia globose to subglobose, longitudinally septate, 14.4–18.2 × 11.4–13.1 μm, usually containing oil drops with four elongated sterigmata, average 2.5 μm width, up to 22 μm long, blunt at the apex. Cystidia absent.

###### Distribution.

REPUBLIC OF KOREA.

###### Additional specimen examined.

REPUBLIC OF KOREA, Gangwon State, Yanggu-gun, Yanggu-eup, Yanggu Arboretum, on a litter, 08 September 2023, SFC20230908-01.

###### Notes.

*Helvellosebacina chrysallidodoma* can be distinguished from other *Helvellosebacina* species by its growth habit of covering the entire plant stem and lacking the paler or thinning margin typical of related species.

###### GenBank accession numbers.

ITS (PV399946, PV399956), LSU (PV399908, PV399918), *rpb*2 (PV417267–PV417268).

##### ﻿ Helvellosebacina koreana

Taxon classificationAnimaliaSebacinalesSebacinaceae

H. Suh, D. Kim & Y.W. Lim sp. nov.

393D9CF9-F5FC-534B-BEF7-C1CBEABDB2A2

858529

Fig. 4

###### Diagnosis.

Basidiomata are fully resupinate on the ground with a smooth and chalky surface.

**Figure 4.**
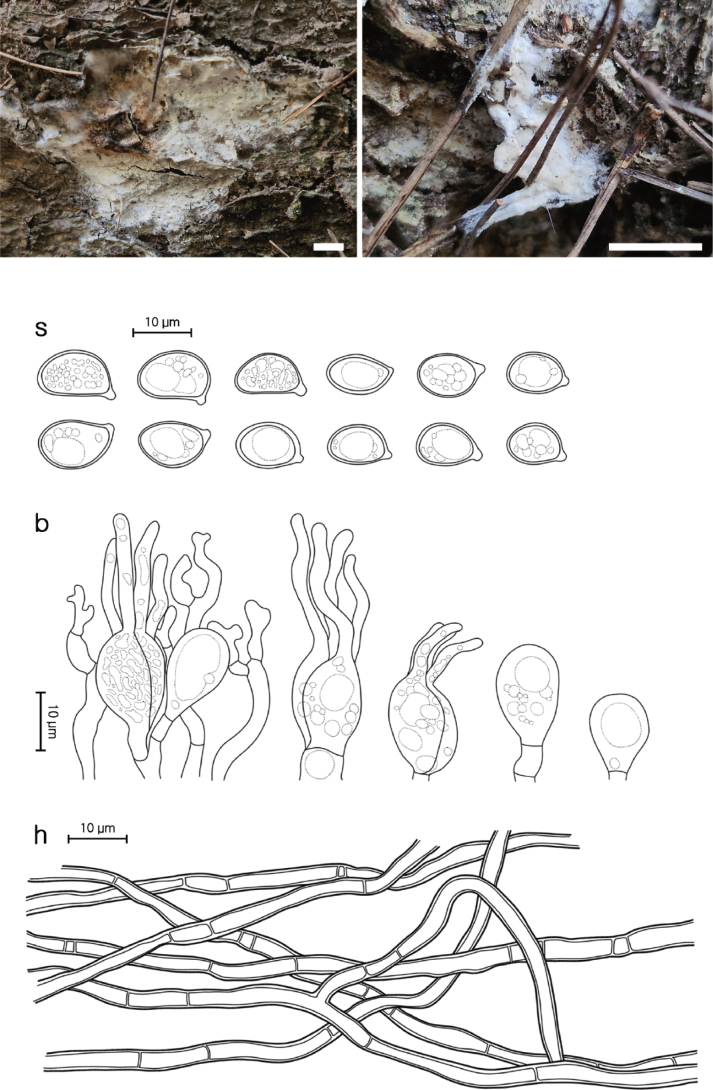
Morphological characters of *Helvellosebacina koreana* (SFC20231005-04, holotype). **A, B** basidiomata; **C** microscopic features, where ‘s’ refers to basidiospores, ‘b’ to basidia and basidioles, and ‘h’ to hyphae. Scale bar: 1 cm (**A, B**).

###### Holotype.

REPUBLIC OF KOREA, Gangwon State, Taebaek-si, Danggol-ro, Mt. Taebaek National Park, on a litter soil under dead *Pinus densiflora*, 05 October 2023, SFC20231005-04 (dried specimen).

###### Etymology.

From the country of origin of the species, the Republic of Korea.

###### Description.

Basidiomata fully resupinate, attached firmly to the substrate, litter, and plant materials on the ground. Surface smooth, dull, chalk-white (5A2) to apricot (5A3), consistency cartilaginous, chalky texture. Margin irregularly thinning out, white to brownish, indistinctly bounded. Basal hyphal system monomitic, subicular hyphae thin-walled up to 0.6 μm, septate with frequent branching, hyaline, without clamp connections, 2.0–3.4 μm thick. Basidiospores elliptical or allantoid, occasionally germinating by repetition, hyaline, smooth, IKI−, CB−, (7.4–)8.2–11.8(–12.7) × (6.1–)6.4–8.4(–8.5) μm (L = 10.2 μm, W = 7.2 μm, Q = 1.4, n = 55/1). Dikaryophyses simple-structured, sparingly ramified, derived from the same hyphae with basidia. Probasidia elliptical to subglobose or pyriform, mature basidia subglobose, longitudinally septate, 14.6–17.0 × 8.0–10.8 μm, usually containing oil drops with four elongated sterigmata, average 2.4 μm width, up to 19 μm long, blunt at the apex. Cystidia absent.

###### Distribution.

REPUBLIC OF KOREA.

###### Additional specimen examined.

REPUBLIC OF KOREA, Gangwon State, Mt. Taebaek National Park, Baekcheon Valley, SFC20231028-04.

###### Notes.

Phylogenetically, *H. koreana* forms a sister clade with two undescribed phylotypes, *Helvellosebacina* pt. 3 (F1143128) and pt. 4 (F1143539), both originating from North Carolina, United States. Compared to *H. chrysallidodoma*, *H. koreana* is observed only as a chalky white residue on the ground, apparently representing an early developmental stage that has not yet formed fully developed basidiomata.

###### GenBank accession numbers.

ITS (PV399955, PV399957), LSU (PV399917, PV399919), *rpb*2 (PV417269–PV417270).

#### ﻿*Sebacina* Tul. & C. Tul., Bot. J. Linn. Soc. 13: 36. 1873.

##### ﻿ Sebacina caducifoliicola

Taxon classificationAnimaliaSebacinalesSebacinaceae

H. Suh, D. Kim & Y.W. Lim sp. nov.

D749D1EF-3CF7-5F3F-85D5-2F2D909CD617

858527

Fig. 5

###### Diagnosis.

*Sebacina caducifoliicola* grows on litter and plant materials lying on the ground, and its tip grows in a frost pattern.

**Figure 5.**
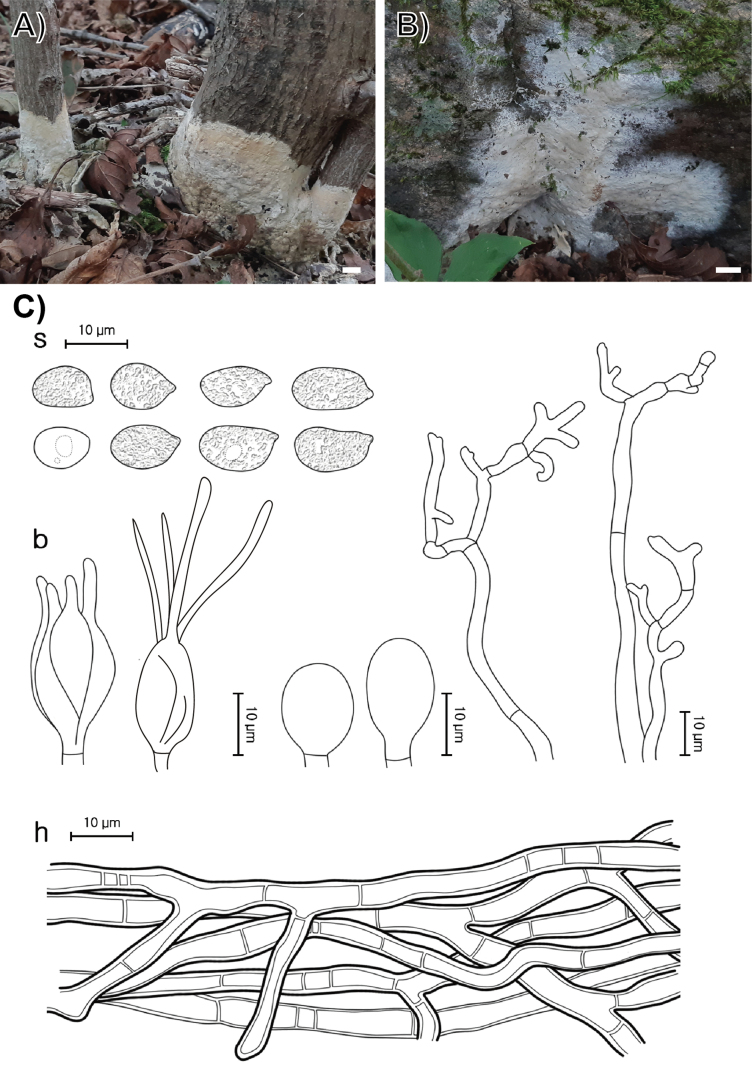
Morphological characters of *Sebacina caducifoliicola* (SFC20230817-41, holotype). **A, B** basidiomata; **C** microscopic features, where ‘s’ refers to basidiospores and resting spores, ‘b’ to basidia, and ‘h’ to hyphae. Scale bar: 1 cm (**A, B**).

###### Holotype.

REPUBLIC OF KOREA, Gangwon State, Yanggu-gun, Yanggu-eup, Yanggu Arboretum, on a litter and plant materials lying on the ground, 17 August 2023. SFC20230817-41 (dried specimen).

###### Etymology.

From *caducifolia*–fallen leaves and *cola*–living in Latin, referring to the basidiomata growing on litter.

###### Description.

Basidiomata fully resupinate, attached firmly to the substrate of litter and plant materials on the ground, growing on soil, forming thick patches several centimeters. Surface smooth, puffy when young, soft to undulate to tuberculate, dull, white (1A1) to yellowish white (4A2), consistency wax-like, cartilaginous when old. Margin growing in a sharp, frost pattern, slightly fibrillose. Basal hyphal system monomitic, hyaline, 2.5–4.0 μm in diameter, septate without clamps, thick-walled up to 1 μm thick. Basidiospores broadly elliptical to oval, hyaline, smooth, IKI−, CB−, (7.6–)8.1–10.0(–10.8) × (5.2–)5.6–6.0(–6.7) μm, with granular contents (L = 9.4 μm, W = 6.5 μm, Q = 1.4, n = 32/2). Resting spores occasionally observed, thick-walled, irregularly shaped. Dikaryophyses coralloid, flagelliformed, and ramified, partially derived from the same hyphae with basidia. Probasidia subglobose or pyriform, mature basidia globose, longitudinally septate, 11.0–13.5 × 9.5–10.5 μm, usually containing oil drops, with four elongated sterigmata, average 2 μm width, up to 24 μm long, blunt at the apex. Cystidia absent.

###### Distribution.

REPUBLIC OF KOREA.

###### Additional specimens examined.

REPUBLIC OF KOREA, Seoul, Gwanak-gu, Mt. Gwanak, on a litter and plant materials lying on the ground, 04 July 2023, SFC20230704-04; REPUBLIC OF KOREA Seoul, Gwanak-gu, Mt.Gwanak, on a litter and plant materials lying on the ground, on a litter and plant materials lying on the ground, 04 July 2023, SFC20240704-05; REPUBLIC OF KOREA, Gangwon State, Yanggu-gun, Yanggu-eup, Yanggu Arboretum, on fallen leaves and litter, 17 August 2023, SFC20230817-45.

###### Notes.

*Sebacina caducifoliicola* is phylogenetically close to *S. pseudocandida*. However, *S. caducifoliicola* can be distinguished from *S. caducifoliicola* by the absence of upright basidiomata, which are present in the latter (Oberwinkler et al. 2014). Furthermore, *S. caducifoliicola* differs from *S. pseudocandida* by 51 nucleotide positions across three genetic regions (ITS: 20 bases; LSU: 11 bases; *rpb*2: 20 bases), supporting its recognition as a distinct species.

###### GenBank accession numbers.

ITS (PV399930– PV399931, PV399940, PV399942), LSU (PV399892–PV399893, PV399902, PV399904), *rpb*2 (PV417273–PV417276).

##### ﻿ Sebacina crystallina

Taxon classificationAnimaliaSebacinalesSebacinaceae

H. Suh, D. Kim & Y.W. Lim sp. nov.

B003EF38-C1A2-5406-AAB9-D4308032645C

858525

Fig. 6

###### Diagnosis.

Basidiomata form sharp and wax-like edges on the substrate.

**Figure 6.**
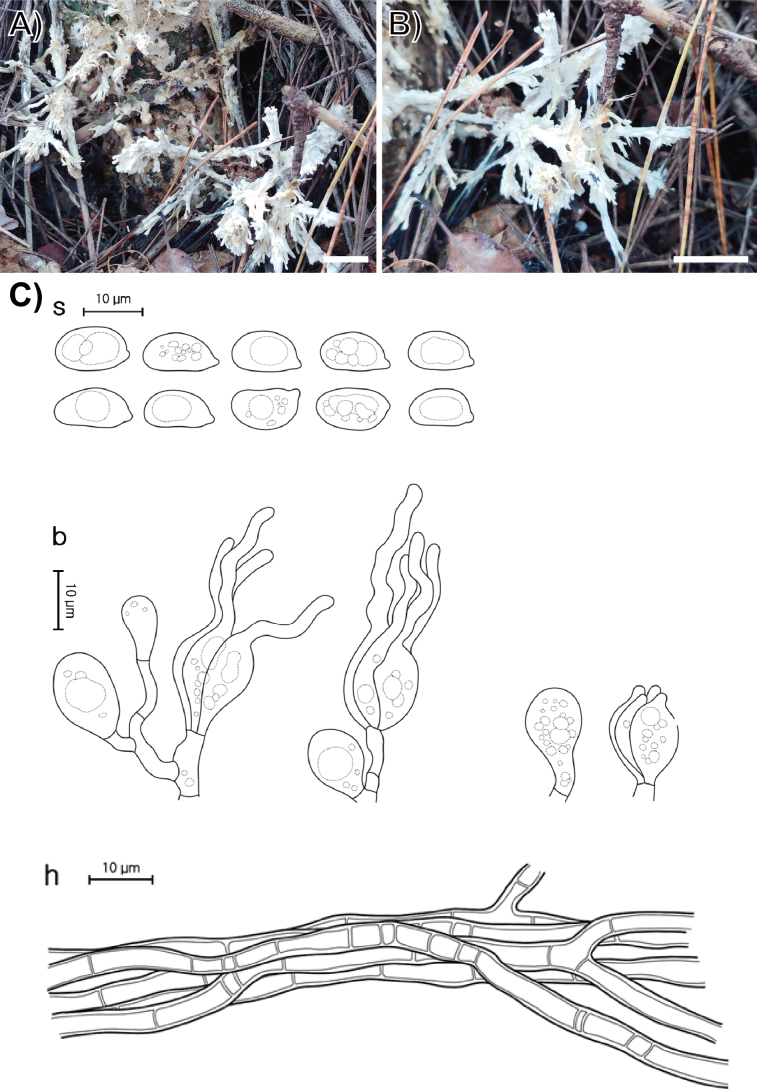
Morphological characters of *Sebacina crystallina* (SFC20200919-11, holotype). **A, B** basidiomata; **C** microscopic features, where ‘s’ refers to basidiospores, ‘b’ to basidia, and ‘h’ to hyphae. Scale bar: 1 cm (**A, B**).

###### Holotype.

REPUBLIC OF KOREA, Gangwon State, Mt. Taebaek National Park, Baekcheon Valley, on dead branches and trunk of the tree, 19 September 2019. SFC20190919-11 (dried specimen).

###### Etymology.

From *crystallus*–crystal in Latin, referring to the crystal-shaped basidiomata.

###### Description.

Basidiomata fully resupinate, attached firmly to the substrate, encrusting small branches and the base of living plants, forming thick patches several centimeters long. Surface smooth to undulate-tuberculate, dull, white (1A1), yellowish white (4A2) to light yellow (4A4) on the center, consistency wax-like, cartilaginous. Margin growing round and distinctly bounded. Basal hyphal system monomitic, hyaline, 2.5–4.5 μm in diameter, septate without clamps, thick-walled up to 0.9 μm thick. Basidiospores broadly elliptical to oval, occasionally germinating by repetition, hyaline, smooth, IKI−, CB−, (7.1–)8.5–12.7(–14.2) × (4.9–)5.2–6.9(–7.2) with granular contents (L = 10.9 μm, W = 5.8 μm, Q = 1.8, n = 57/2). Dikaryophyses simple-structured, flagelliformed, derived from the same hyphae with basidia. Probasidia elliptical to pyriform, usually with lateral hyphal branches, mature basidia subglobose to pyriform, longitudinally septate, 13.1–14.1 × 9.2–10.6 μm on average, usually containing oil drops, with four elongated sterigmata, average 3 μm width, up to 36 μm long, blunt to tapered at the apex. Cystidia absent.

###### Distribution.

REPUBLIC OF KOREA, RUSSIAN FEDERATION.

###### Additional specimens examined.

RUSSIAN FEDERATION, Primorskiy kray, Vitiaz’ settlement, Gamov Peninsula, on a plant litter, 16 August 2017, NIBRFG0000504700; REPUBLIC OF KOREA, Seoul, Gwanak-gu, Seoul National University, on a trunk of a tree, 29 July 2023, SFC20230729-09.

###### Notes.

*Sebacina crystallina* is phylogenetically closely related to *Sebacina schweinitzii*. Morphologically, the two species share spathulate to clavarioid basidiomata and thick-walled hyphae. However, they can be distinguished by their hymenial surfaces: *S. crystallina* exhibits an opaque, dull, white (1A1) hymenium, whereas *S. schweinitzii* has a transparent, smooth surface (Oberwinkler et al. 2014). The two species also exhibit regional differences; *S. crystallina* is found in East Asia, whereas *S. schweinitzii* occurs in Europe.

###### GenBank accession numbers.

ITS (PV399921, PV399927, PV399934), LSU (PV399884, PV399890, PV399896), *rpb*2 (PV417271–PV417272).

##### ﻿ Sebacina incarnata

Taxon classificationAnimaliaSebacinalesSebacinaceae

H. Suh, D. Kim & Y.W. Lim sp. nov.

7139CD20-4952-50B6-9134-5A6F78A0CFA8

858526

Fig. 7

###### Diagnosis.

Basidiomata surface exhibits pale orange to pastel red.

**Figure 7.**
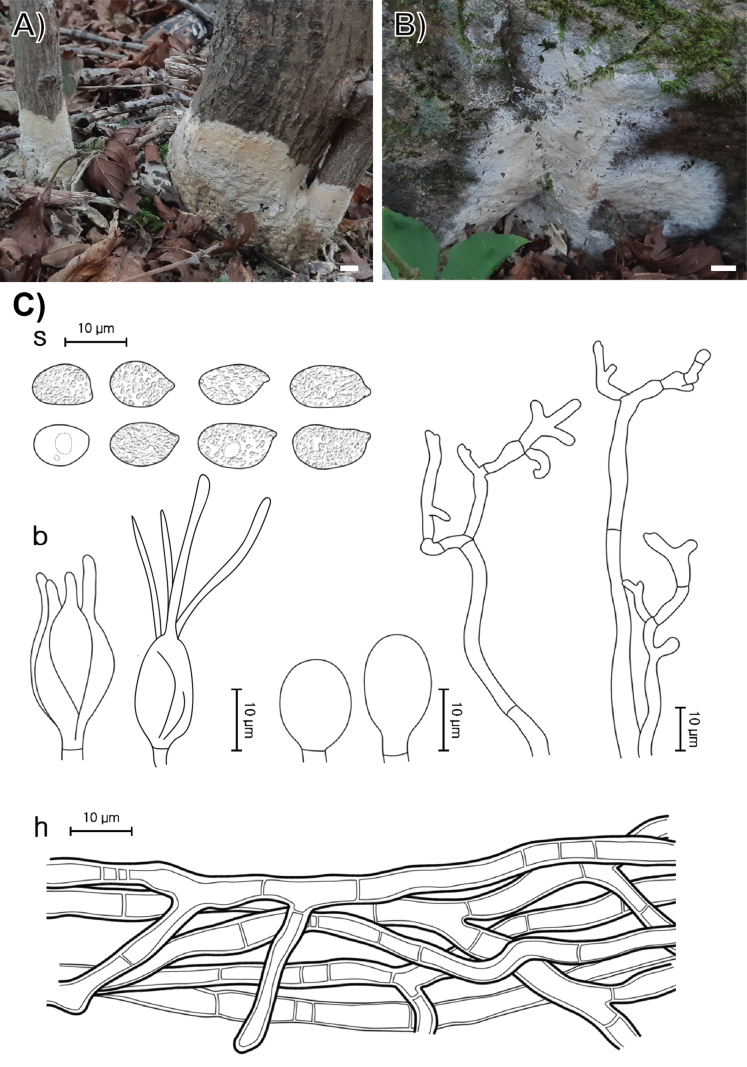
Morphological characters of *Sebacina incarnata*. **A** basidiomata (SFC20200821-21, holotype); **B** basidiomata (SFC20200821-26); **C** microscopic features, where ‘s’ refers to basidiospores and resting spores, ‘b’ to basidia and dikaryophyses, and ‘h’ to hyphae. Scale bar: 1 cm (**A, B**).

###### Holotype.

REPUBLIC OF KOREA, Gangwon State, Jeongseon-gun, Gohan-eup, Gohan-ri, 214-25, on a trunk of a deciduous tree, 21 August 2020, SFC20200821-21 (dried specimen).

###### Etymology.

From *incarnata*–flesh-colored in Latin, referring to the whitish-red color of the basidiomata.

###### Description.

Basidiomata fully resupinate, attached firmly to the substrate, trunks of Acer and cork oak, forming thick wax-like patches on the substrate, spanning several centimeters. Surface smooth, dull, pale orange (6A3) to pastel red (7A3), consistency wax-like, cartilaginous, rough, and nodulous texture. Margin irregularly thinning out, white to brownish, distinctly bounded. Basal hyphal system monomitic, subicular hyphae thick-walled up to 0.8 μm, septate with frequent branching, hyaline, without clamp connections, 2.8–4.2 μm thick. Basidiospores elliptical or allantoid, hyaline, smooth, IKI−, CB−, (8.6–)9.2–13.0(–13.2) × (5.7–)6.0–8.0(–8.6) μm with granular contents (L = 11.0 μm, W = 7.0 μm, Q = 1.57, n = 43/2). Resting spores occasionally observed, thick-walled, irregularly shaped, measuring less than 10 μm in diameter. Dikaryophyses coralloid, flagelliformed, and strongly ramified, mostly derived from the same hyphae with basidia. Probasidia elliptical to subglobose, mature basidia subglobose, longitudinally septate, 15.6–21.1 × 12.0–14.4 μm, usually containing oil drops, with four elongated sterigmata, average 2.0 μm width, up to 22 μm long, tapered at the apex. Cystidia absent.

###### Distribution.

REPUBLIC OF KOREA.

###### Additional specimen examined.

REPUBLIC OF KOREA, Gangwon State, Jeongseon-gun, Gohan-eup, Gohan-ri, 214-25, on a rock and litter, 21 August 2020, SFC20200821-26;

###### Notes.

*Sebacina incarnata* is phylogenetically close to *S. aciculicola*. Both species exhibit a nodulous surface at maturity but can be distinguished by their color when fresh: *S. incarnata* is pastel red (7A3), whereas *S. aciculicola* is white to cream (Dong et al. 2025).

###### GenBank accession numbers.

ITS (PV399928–PV399929), LSU (PV399891).

##### ﻿ Sebacina orientalis

Taxon classificationAnimaliaSebacinalesSebacinaceae

H. Suh, D. Kim & Y.W. Lim sp. nov.

A650C138-AE92-5B80-9DAA-63697A9EBDA8

858528

Fig. 8

###### Diagnosis.

Basidiomata mainly develop on dead branches. The basidiomata surface is white to milk-white with yellowish spots.

**Figure 8.**
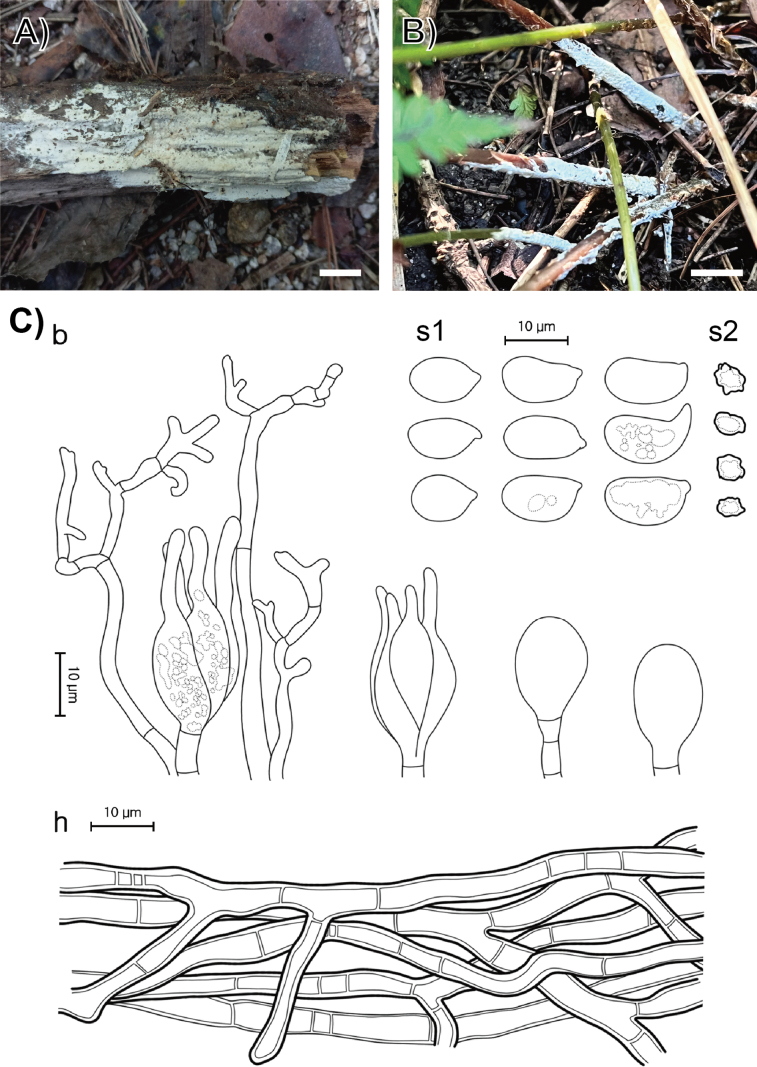
Morphological characters of *Sebacina orientalis*. **A** basidiomata (SFC20230908-13, holotype); **B** basidiomata (SFC20230908-04); **C** microscopic features, where ‘b’ refers to basidia, ‘s’ to basidiospores and resting spores, and ‘h’ to hyphae. Scale bar: 1 cm (**A, B**).

###### Holotype.

REPUBLIC OF KOREA, Gangwon State, Yanggu-gun, Yanggu-eup, Yanggu Arboretum, on a dead branch, 08 September 2023. SFC20230908-13 (dried specimen).

###### Etymology.

From *orientalis*–eastern in Latin, referring to the eastern origin of the species.

###### Description.

Basidiomata fully resupinate, attached firmly to the substrate, young basidiomata growing on stems of shrubs and dead branches, encrusting everywhere when old, forming thin patches on the substrate. Surface smooth, dull, white (1A1) to yellowish white (4A2), consistency wax-like, cartilaginous. Margin irregularly thinning out and distinctly bounded. Basal hyphal system monomitic, subicular hyphae thick-walled, septate with frequent branching, hyaline, without clamp connections, 3.0–4.3 μm thick. Basidiospores elliptical or allantoid, hyaline, smooth, IKI−, CB−, (7.1–)9.0–11.6(–12.6) × (5.8–)6.1–7.6(–8.5) μm with granular contents (L = 10.3 μm, W = 7.1 μm, Q = 1.4, n = 31/2). Resting spores usually observed, thick-walled, irregularly shaped. Dikaryophyses coralloid, flagelliformed, and tinily ramified, derived from the same hyphae with basidia. Probasidia globose to subglobose, mature basidia elliptical or pyriform, longitudinally septate, 13.1–17.3 × 7.5–11.8 μm, usually containing oil drops, with elongated four sterigmata, average 2.5 μm width, up to 16.0 μm long, blunt at the apex. Cystidia absent.

###### Distribution.

REPUBLIC OF KOREA.

###### Additional specimens examined.

REPUBLIC OF KOREA, Gangwon State, Yanggu-gun, Gwangchi National Recreation Forest, on a tree, 16 August 2023, SFC20230816-55; REPUBLIC OF KOREA, Gangwon State, Yanggu-gun, Yanggu-eup, Yanggu Arboretum, on a rotten leave, 17 August 2023, SFC20230817-43; Republic of Korea, Gangwon State, Yanggu-gun, Gwangchi National Recreation Forest on a rotten branch, 07 September 2023, SFC20230907-02; REPUBLIC OF KOREA, Gangwon State, Yanggu-gun, Gwangchi National Recreation Forest, on a rotten branch, 07 September 2023, SFC20230907-03; REPUBLIC OF KOREA, Gangwon State, Yanggu-gun, Yanggu-eup, Yanggu Arboretum, on a rotten rotten leave, 08 September 2023, SFC20230908-04; REPUBLIC OF KOREA, Gangwon State, Yanggu-gun, Yanggu-eup, Yanggu Arboretum, on a rotten leave, 08 September 2023, SFC20230908-05; REPUBLIC OF KOREA, Gangwon State, Yanggu-gun, Yanggu-eup, Yanggu Arboretum, on a rotten leave, 08 September 2023, SFC20230908-19.

###### Notes.

*Sebacina orientalis* is phylogenetically close to *S. incarnata*. The two species can be distinguished by the color of the basidiomata surface: *S. orientalis* is white (1A1) to yellowish white (4A2), whereas *S. incarnata* is pale orange (6A3) to pastel red (7A3).

###### GenBank accession numbers.

ITS (PV399937, PV399941, PV399944–PV399945, PV399948–PV399950, PV399952–PV399953), LSU (PV399899, PV399903, PV399906–PV399907, PV399910–PV399912, PV399914–PV399915), *rpb*2 (PV417258–PV417262).

#### ﻿New to Korea

##### ﻿ Sebacina aciculicola

Taxon classificationAnimaliaSebacinalesSebacinaceae

J.H. Dong, Xin Zhang bis, Y.C. Dai & F. Wu, MycoKeys 118: 112. 2025.

5E9E12B0-9E31-5101-AE23-126BA4E4A6DA

857598

Fig. 9

###### Notes.

Korean collections of *Sebacina aciculicola* largely agree with the original description from Dong et al. (2025), exhibiting an oriaceous color and appearing white to cream when fresh. However, Korean specimens form thick, nodulose basidiomata, turning sand (4A3) to greyish yellow (1B4) to white (1A1) towards the margin.

**Figure 9.**
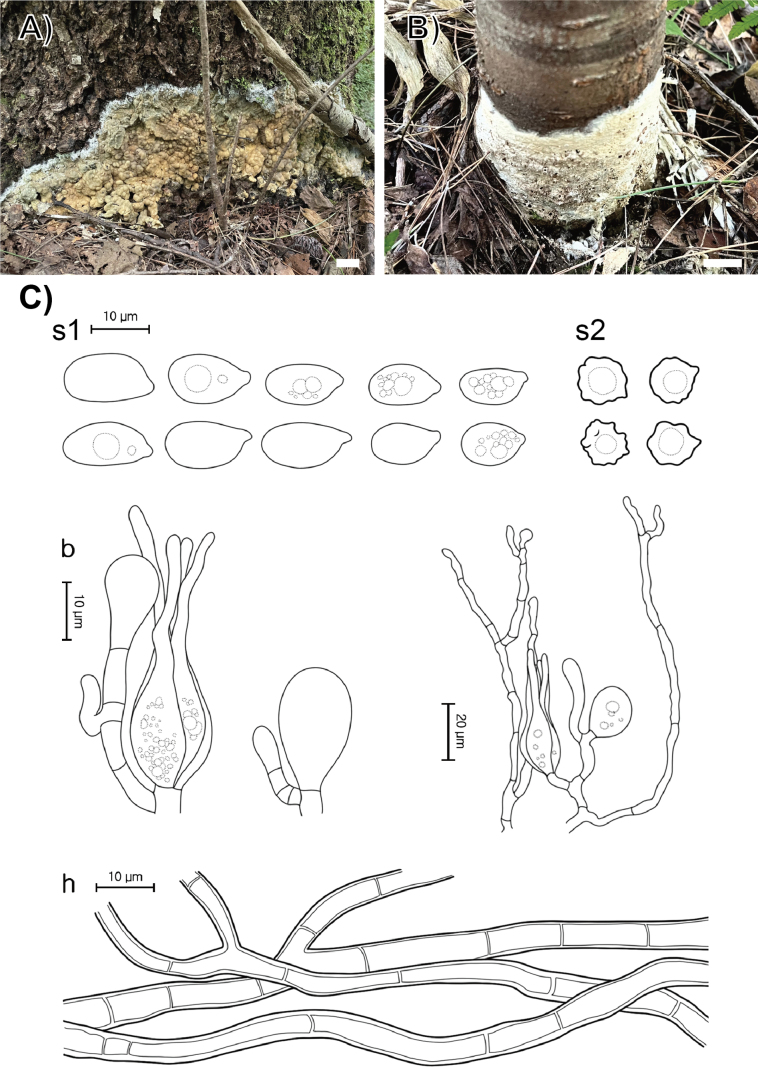
Morphological characters of *Sebacina aciculicola*. **A** basidiomata (SFC20230817-46, holotype); **B** basidiomata (SFC20230908-06); **C** microscopic features, where ‘s’ refers to basidiospores and resting spores, ‘b’ to basidia, basidioles, and dikaryophyses, and ‘h’ to hyphae. Scale bar: 1 cm (**A, B**).

###### Distribution.

ASIA.

###### Additional specimens examined.

REPUBLIC OF KOREA, Gyeongsangbuk-do, Seongju-gun, Gacheon-myeon, Gaya National Park, on a trunk of a deciduous tree, 20 August 2020, NIBRFG0000508545; REPUBLIC OF KOREA, Seoul, Gwanak-gu, Seoul National University, on a trunk of a living tree, 01 August 2023, SFC20230802-01; REPUBLIC OF KOREA, Gyeongsangbuk-do, Seongju-gun, Hakgasan Natural Recreation Forest, on a living tree, 08 September 2023, SFC20230908-06.

###### GenBank accession numbers.

ITS (PV399923, PV399935, PV399943, PV399951), LSU (PV399886, PV399897, PV399905, PV399913), *rpb*2 (PV417255–PV417257).

##### ﻿ Sebacina ocreata

Taxon classificationAnimaliaSebacinalesSebacinaceae

(Berk.) Oberw., Garnica & K. Riess, Mycological Progress 13 (3): 468. 2014.

A4CA8DCD-ADCE-5DBC-A2F2-9A393133BFA8

808196

Fig. 10

= Thelephora ocreata Berk., Hooker’s Journal of Botany and Kew Garden Miscellany 8:239 (1856).
= Tremellodendron ocreatum (Berk) P. Roberts, Mycotaxon 89 (2): 434 (2004).

###### Notes.

Korean collections of *Sebacina ocreata* largely agree with the original description by Berkeley (1856), exhibiting fully resupinate basidiomata that are firmly attached to herbaceous plants. They form thick, wax-like patches covering the entire plant stem, with a smooth, dull, white (1A1) surface and a cartilaginous consistency. However, Korean specimens do not grow upright with acute tips. They also differ in micromorphology, having larger basidiospores (10.7–13.1 × 8.0–9.1 μm) compared to the original description (7–10 × 4–5.5 μm). In comparison with the specimen from Guyana (Henkel et al. 2004), the hymenium of the Korean collections does not turn dark grey when fresh.

**Figure 10.**
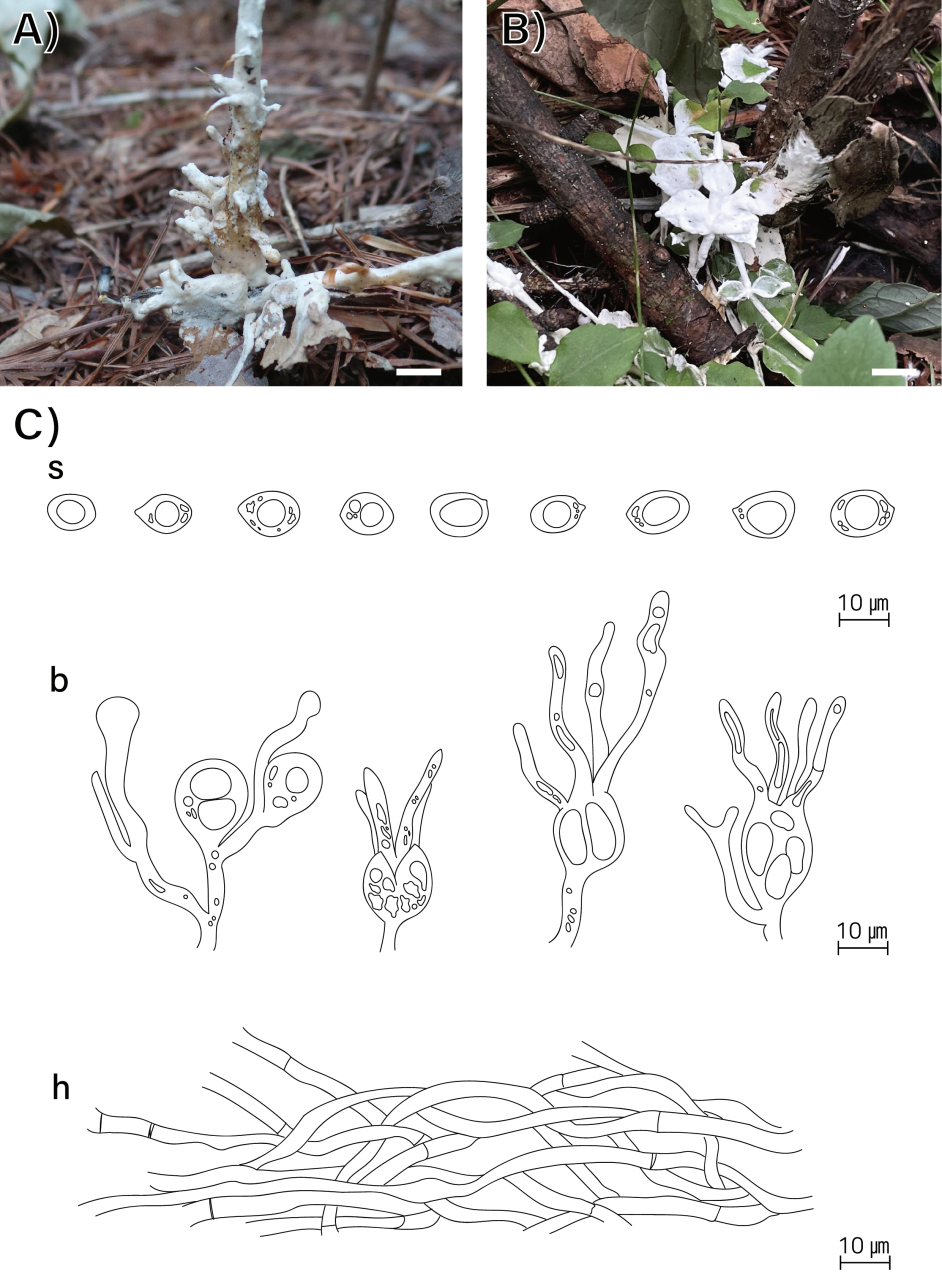
Morphological characters of *Sebacina ocreata*. **A** basidiomata (SFC20130917-H16); **B** basidiomata (SFC20230817-39); **C** microscopic features, where ‘b’ refers to basidia, ‘s’ to basidiospores, and ‘h’ to hyphae. Scale bar: 1 cm (**A, B**).

###### Distribution.

ASIA, EUROPE, SOUTH AMERICA.

###### Additional specimens examined.

REPUBLIC OF KOREA, Gyeongsangbuk-do, Mt. Hakkasan, 17 September 2013, on a plant stem, SFC20130917-H16; REPUBLIC OF KOREA, Jeju, Gotjawal Forest, Dongbaek Dongsan, 01 July 2015, on a litter plant stem, SFC20150701-21; REPUBLIC OF KOREA, Seoul, Gwanak-gu, Seoul National University, 22 July 2023, on a plant stem, SFC20230722-33; REPUBLIC OF KOREA, Gangwon State, Yanggu-gun, Gwangchi National Recreation Forest, on a plant stem, 16 August 2023, SFC20230816-52; REPUBLIC OF KOREA, Gangwon State, Yanggu-gun, Yanggu-eup, Yanggu Arboretum, on a plant stem and leaf, 17 August 2023, SFC20230817-39; REPUBLIC OF KOREA, Gangwon State, Yanggu-gun, Yanggu-eup, Yanggu Arboretum, on a plant stem, 8 September 2023, SFC20230908-02.

###### GenBank accession numbers.

ITS (PV399924–PV399925, PV399933, PV399936, PV399939, PV399947), LSU (PV399887–PV399888, PV399895, PV399898, PV399901, PV399909), *rpb*2 (PV417251–PV417254).

## ﻿Discussion

Crust-like *Sebacinaceae* species have been reported in numerous environmental samples (Schmidt et al. 2013; Abarenkov et al. 2024). However, their taxonomy remains understudied because of the inconspicuous morphology of basidiomata. To date, only seven genera and 28 species of *Sebacinaceae* have legitimate sequence data accompanied by morphological descriptions (Oberwinkler et al. 2014; Kirschner et al. 2017; Sesli 2021; Dong et al. 2025). Moreover, a large portion of *Sebacina* sequences deposited in the GenBank database remains unidentified at the species level (3,690 as “uncultured *Sebacina*” and 727 as “*Sebacina* sp.”), with only a limited number reliably assigned to described species (Benson et al. 2012; GenBank, accessed 11 June 2025, at https://www.ncbi.nlm.nih.gov/).

Additionally, several misassigned taxonomic names were identified in public databases, including *S. epigaea* and *S. incrustans*. These sequences were re-examined using a multigene phylogeny and reassigned accordingly. For example, sequences previously identified as *S. epigaea* were reassigned to *Sebacina* pt. 17 (TUB 020005) and *Sebacina* pt. 18 (TUB 019999 and TUB 020017), whereas those labeled as *S. incrustans* (TUB 019637 and TUB 020011) were reassigned to *S. ocreata*. Such misidentifications are particularly frequent among morphologically similar taxa, resulting in a substantial number of inaccurately labeled sequences in public databases (Cho et al. 2023). These revisions highlight the need for curation and correction of public sequence databases to ensure more accurate taxonomic classification. To resolve species boundaries within crust-like *Sebacinaceae*, we employed multiple genetic markers, including ITS, LSU, and *rpb*2. However, separation between the genera *Sebacina* and *Helvellosebacina* still receives limited support, consistent with previous studies (Moyersoen and Weiß 2014; Oberwinkler et al. 2014). This weak resolution may reflect the insufficient number of species with complete three-marker datasets. Expanding the sampling of basidiomata with full marker coverage and incorporating additional loci could improve phylogenetic resolution of genus-level boundaries within crust-like *Sebacinaceae*. The newly proposed species lacked distinct morphological features to differentiate them from one another, making it difficult to present reliable diagnostic characters. This morphological ambiguity also hindered the establishment of complete three-marker datasets from basidiomata collections. Therefore, ongoing efforts should prioritize the generation of full multigene datasets together with the identification of stable morphological and ecological characteristics that can aid in the reliable delimitation of crust-like *Sebacinaceae* species.

The integrated analysis revealed unexpectedly high species richness of crust-like *Sebacinaceae* in Korea. Although only a single species (*Sebacina incrustans*) had been previously reported in Korea (Lee et al. 2015), this study uncovered 11 distinct species through basidiomata sampling and 31 phylotypes when metabarcoding data were included. This result emphasizes the high degree of morphological similarity among crust-like *Sebacinaceae* species and demonstrates the effectiveness of molecular methods in distinguishing these taxa. The metabarcoding approach also revealed substantial diversity of root-associated crust-like *Sebacinaceae* species, consistent with previous findings based on soil DNA sequencing (Landínez-Torres et al. 2019). Given that our analysis was limited to the roots of only two ectomycorrhizal tree species (*Pinus* and *Quercus*), expanding the survey to a broader range of ectomycorrhizal host plants is likely to reveal even greater diversity. Furthermore, *Serendipita*, a genus within *Sebacinales*, has been reported to act as an endophyte in herbaceous crops and non-ectomycorrhizal plants (Riess et al. 2014), indicating that broader sampling, including non-ectomycorrhizal plants, could uncover a wider range of crust-like *Sebacinaceae* species and their ecological interactions.

Most Asian phylotypes identified in this study appear to represent previously undocumented lineages. The low overlap with European and American species supports earlier findings that ectomycorrhizal fungi exhibit strong biogeographic structuring, likely shaped by host plant and geographic specificity. Many cases have been reported in which morphologically similar species in other ectomycorrhizal genera, such as *Amanita*, *Laccaria*, and *Lactarius*, have been identified as distinct species on different continents (Wisitrassameewong et al. 2016; Cho et al. 2018; Cui et al. 2018; Lee et al. 2019). In line with this pattern, *S. incrustans*, a well-known European ectomycorrhizal species (Tulasne and Tulasne 1871), was not detected in our Korean samples. Instead, closely related species (*S. aciculicola*, *S. incarnata*, *S. orientalis*) were found exclusively in Asia, suggesting regional endemism. Notably, only three phylotypes matched known species from other continents—*S. dimitica* and *S. epigaea* from Europe (Weiß and Oberwinkler 2001; Riess et al. 2013; Oberwinkler et al. 2014) and *S. ocreata* from Europe and South America (Selosse et al. 2002; Henkel et al. 2004; Riess et al. 2013). The presence of such globally distributed species appears to be exceptional. Interestingly, these three species were detected in association with both *Pinus* and *Quercus*, suggesting that their broad host range may contribute to their widespread geographic distribution. Given the presence of locally endemic crust-like *Sebacinaceae* within various clades, we predict that global crust-like *Sebacinaceae* diversity remains vastly underestimated, particularly in regions that remain largely unexplored.

A remarkable aspect of this study is the minimal overlap between crust-like *Sebacinaceae* taxa detected through basidiomata sampling and root-associated metabarcoding. Among the 31 phylotypes detected, only three species (*S. aciculicola*, *S. orientalis*, and *S. ocreata*) were present in both datasets. This discrepancy may be attributed to several factors, including (1) limited sampling of basidiomata, (2) cryptic or non-fruiting lifestyles of many crust-like *Sebacinaceae* taxa, consistent with previous reports of species acting as endophytes or forming orchid mycorrhiza (Weiß et al. 2011), and (3) local environmental conditions not conducive to basidioma formation. For instance, *S. epigaea* and *S. dimitica* were frequently detected in root data but were never observed as basidiomata in Korea. However, *S. epigaea* has been reported based on basidiomata data from Europe (Oberwinkler et al. 2014), which may support the first and third factors. Broader spatial sampling will be necessary to bridge this gap.

Beyond taxonomic resolution, metabarcoding also provided insights into the ecological behavior of crust-like *Sebacinaceae*. Basidiomata were typically found on stems or leaves of herbaceous plants (Oberwinkler et al. 2014; Sesli 2021) and rarely at the base of *Pinus* or *Quercus* trees. In contrast, root-associated crust-like *Sebacinaceae* phylotypes were common in both hosts, and most species produced basidiomata in locations not directly associated with either host tree. This disparity suggests that basidioma occurrence may not always reflect the true ecological host range of crust-like *Sebacinaceae* species. Our findings support the view that crust-like *Sebacinaceae* members are generally host-preferential rather than strictly host-specific (Tedersoo et al. 2014; Schön et al. 2022). Nevertheless, further clarification of host associations will require comprehensive root sampling across diverse plant groups.

## ﻿Conclusion

This study provides a comprehensive assessment of crust-like *Sebacinaceae* in Asia, revealing substantial hidden diversity and describing six new species within *Sebacinaceae*. By combining multigene phylogenetic analysis and root-associated metabarcoding, we uncovered numerous previously undescribed crust-like *Sebacinaceae* lineages and documented their host associations. Despite this progress, many phylotypes remain unlinked to identifiable basidiomata, highlighting the need for continued integrative efforts. Addressing these gaps will require expanding molecular marker datasets to resolve undesignated phylotypes, as well as investigating the ecological roles and biogeographic patterns of crust-like *Sebacinaceae* across a broader range of habitats and hosts.

## Supplementary Material

XML Treatment for Helvellosebacina chrysallidodoma

XML Treatment for Helvellosebacina koreana

XML Treatment for Sebacina caducifoliicola

XML Treatment for Sebacina crystallina

XML Treatment for Sebacina incarnata

XML Treatment for Sebacina orientalis

XML Treatment for Sebacina aciculicola

XML Treatment for Sebacina ocreata

## ﻿Additional information

### Use of AI

AI is used to refine and clarify the sentences and received assistance in writing code for visualization.

### Adherence to national and international regulations

All the fungal strains used in this study have been legally obtained, respecting the Convention on Biological Diversity (Rio Convention).

### Author ORCIDs

Hannah Suh https://orcid.org/0009-0003-7526-1731

Chang Wan Seo https://orcid.org/0000-0002-5948-1836

Ki Hyeong Park https://orcid.org/0000-0002-8832-7643

Shinnam Yoo https://orcid.org/0000-0003-3686-3759

Dohye Kim https://orcid.org/0009-0007-0551-099X

Yoonhee Cho https://orcid.org/0000-0002-0743-0914

Young Woon Lim https://orcid.org/0000-0003-2864-3449

### Data availability

GenBank accessions for basidiomes: ITSPV399920–PV399957; LSUPV399883–PV399919; rpb2 PV417251–PV417276. GenBank accessions for root tip metabarcoding data: ITSPV422624–PV422663; LSUPV422665–PV422704. Raw metabarcoding data used in this study are available at the NCBI Sequence Read Archive (https://www.ncbi.nlm.nih.gov/) under BioProject ID PRJNA894948.

## Supplementary materials

Supplementary material 1 Additional information Data type docx https://binary.pensoft.net/file/1511944This dataset is made available under the Open Database License (http://opendatacommons.org/licenses/odbl/1.0/). The Open Database License (ODbL) is a license agreement intended to allow users to freely share, modify, and use this Dataset while maintaining this same freedom for others, provided that the original source and author(s) are credited.Hannah Suh, Chang Wan Seo, Ki Hyeong Park, Shinnam Yoo, Dohye Kim, Yoonhee Cho, Young Woon Lim

Supplementary material 2 Alignment file of *Sebacinaceae* Data type fasta Explanation note Alignment file of *Sebacinaceae* phylogenetic tree used in Fig. 2. https://binary.pensoft.net/file/1511945This dataset is made available under the Open Database License (http://opendatacommons.org/licenses/odbl/1.0/). The Open Database License (ODbL) is a license agreement intended to allow users to freely share, modify, and use this Dataset while maintaining this same freedom for others, provided that the original source and author(s) are credited.Hannah Suh, Chang Wan Seo, Ki Hyeong Park, Shinnam Yoo, Dohye Kim, Yoonhee Cho, Young Woon Lim
