# Corrigendum: Suh H, Seo CW, Park KH, Yoo S, Kim D, Cho Y, Lim YW (2026) Hidden diversity of crust-like *Sebacinaceae* (*Sebacinales*, *Agaricomycetes*) in Asia. IMA Fungus 17: e168486. https://doi.org/10.3897/imafungus.17.168486

**DOI:** 10.3897/imafungus.17.187535

**Published:** 2026-02-26

**Authors:** Hannah Suh, Chang Wan Seo, Ki Hyeong Park, Shinnam Yoo, Dohye Kim, Yoonhee Cho, Young Woon Lim

**Affiliations:** 1 School of Biological Sciences and Institute of Biodiversity, Seoul National University, Seoul, Republic of Korea Swedish University of Agricultural Sciences Uppsala Sweden; 2 Forest Entomology and Pathology Division, National Institute of Forest Science, Seoul, Republic of Korea Seoul National University Seoul Republic of Korea; 3 Department of Forest Biomaterials and Technology, Swedish University of Agricultural Sciences, Uppsala, Sweden Forest Entomology and Pathology Division, National Institute of Forest Science Seoul Republic of Korea

After reviewing the paper recently published by Suh et al. (2025), we noticed that the figures included are not the final versions. We therefore provide below the corrected figures with the accurate information. Additionally, errors identified in the manuscript, including certain references, the spelling of an author's name with a special character (Weiß), and funding information, have been corrected accordingly. We sincerely apologize for the oversight in the final review process.

**Figure 1.**
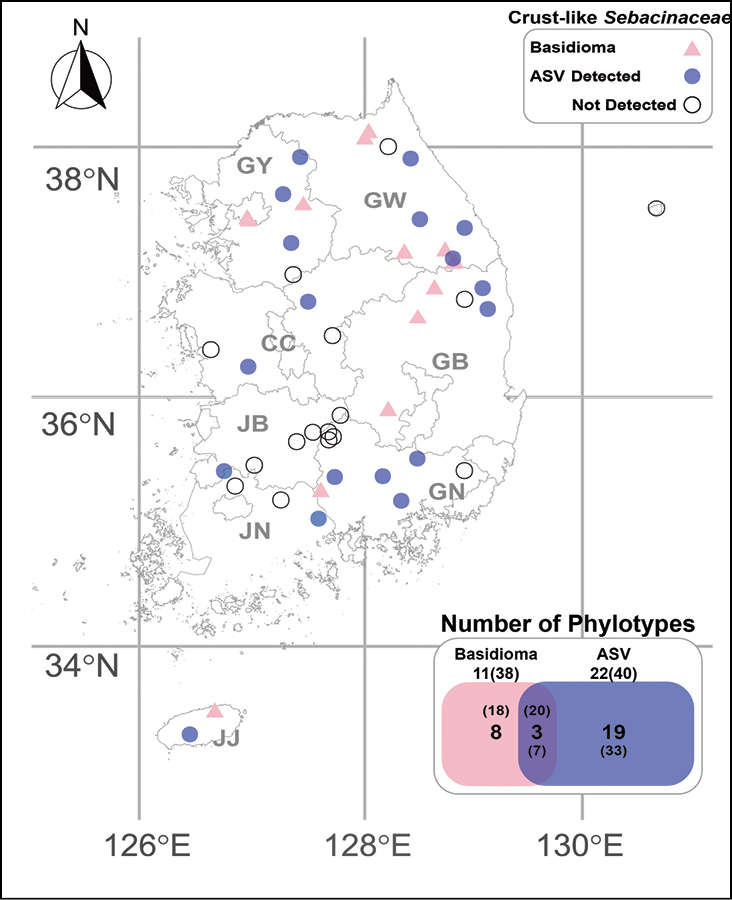
An overview of *Sebacinaceae* sampling sites and sample type comparison in Korea. Sampling sites include basidiomata (pink), root-associated metabarcoding data collection sites where crust-like *Sebacinaceae* were detected (blue), and sites where crust-like *Sebacinaceae* taxa were not detected (empty). The sample type comparison shown in the box indicates the number of crust-like *Sebacinaceae* taxa identified from basidiomata and root-associated metabarcoding data (ASV). Bold numbers indicate the number of each phylotype, and numbers in brackets represent the number of specimens or ASV counts.

**Figure 2.**
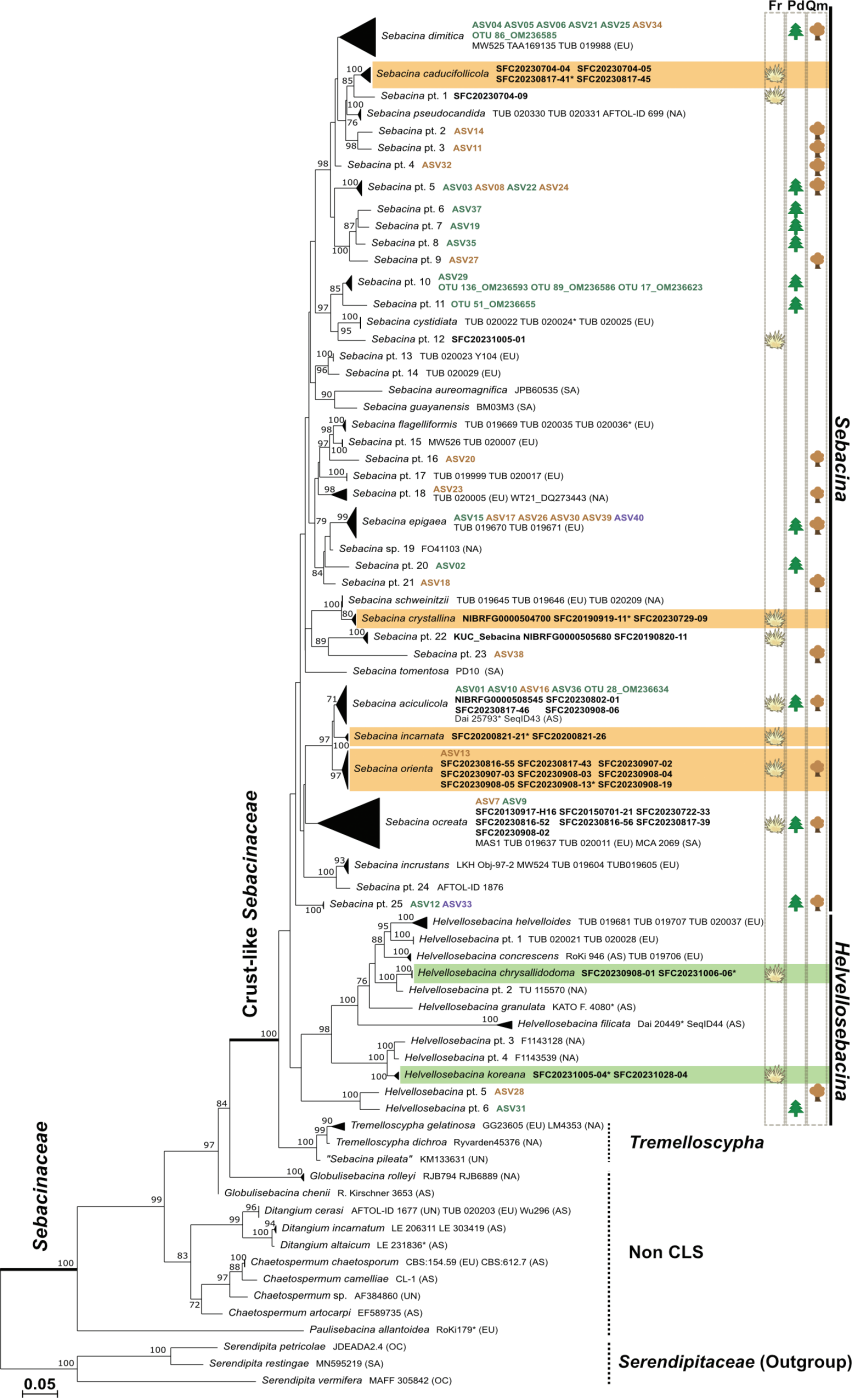
Phylogenetic tree inferred from maximum likelihood (ML) analysis based on a sequence of ITS, LSU, and *rpb2* sequences for the family *Sebacinaceae* and outgroups. ML bootstrap support values above 70 are shown at the nodes. Specimen voucher or strain numbers are given after each species name. The continental origin of each specimen is indicated in brackets: AS (Asia), EU (Europe), NA (North America), OC (Oceania), SA (South America), and UN (unknown). *Sebacina* pt. refers to phylotype data consisting of unknown species based on basidiomataor metabarcoding data. New species are highlighted in orange for *Sebacina* and green for *Helvellosebacina*. Basidiomata from this study are in bold, with a yellow crust-like symbol on the right. Metabarcoding data from Korea are bolded in green for *Pinus densiflora*, brown for *Quercus* spp., and purple for samples associated with both hosts. Green and brown host tree symbols on the right indicate *Pinus densiflora* and *Quercus* spp., respectively. Specimens marked with an asterisk (*) indicate holotypes.

**Figure 4.**
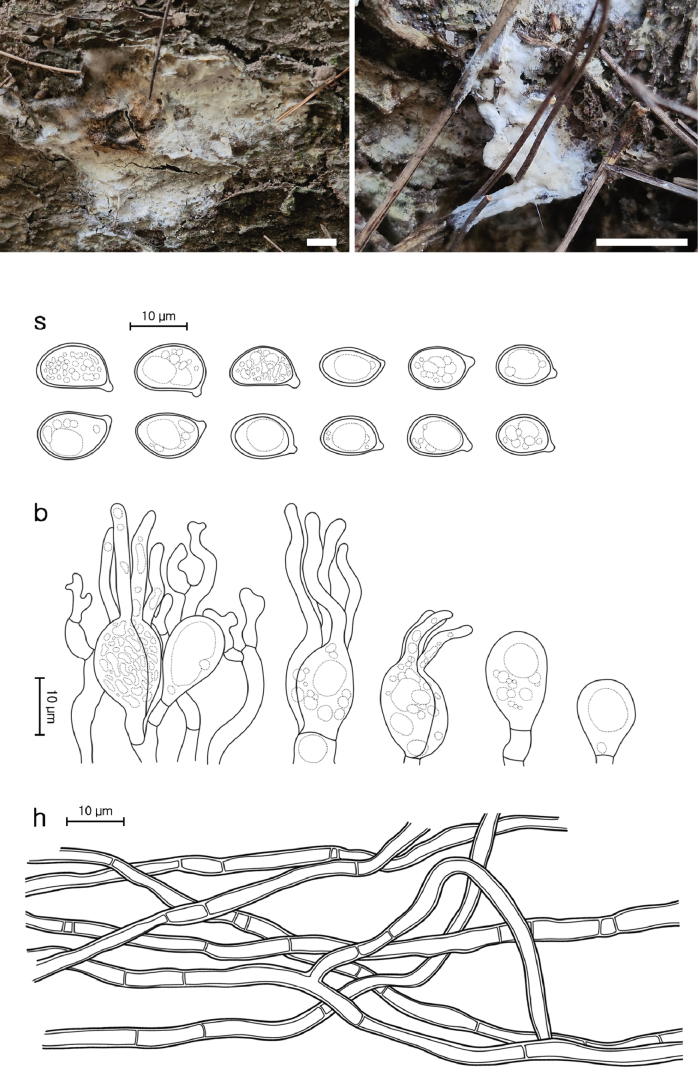
Morphological characters of *Helvellosebacina koreana* (SFC20231005-04, holotype). **A, B** basidiomata; **C** microscopic features, where ‘s’ refers to basidiospores, ‘b’ to basidia, basidioles, and dikaryophyses, and ‘h’ to hyphae. Scale bars: 1 cm (**A, B**).

**Figure 5.**
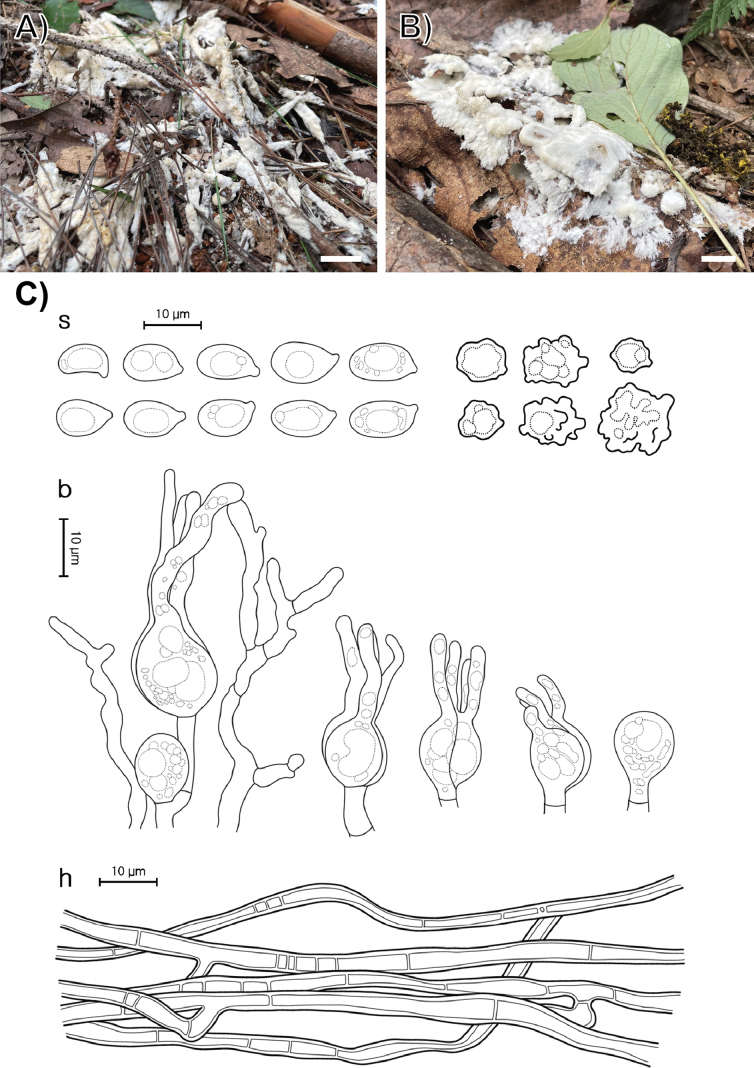
Morphological characters of *Sebacina caducifoliicola* (SFC20230817-41, holotype). **A, B** basidiomata; **C** microscopic features, where ‘s’ refers to basidiospores and resting spores, ‘b’ to basidioles, and dikaryophyses, and ‘h’ to hyphae. Scale bars: 1 cm (**A, B**).

**Figure 6.**
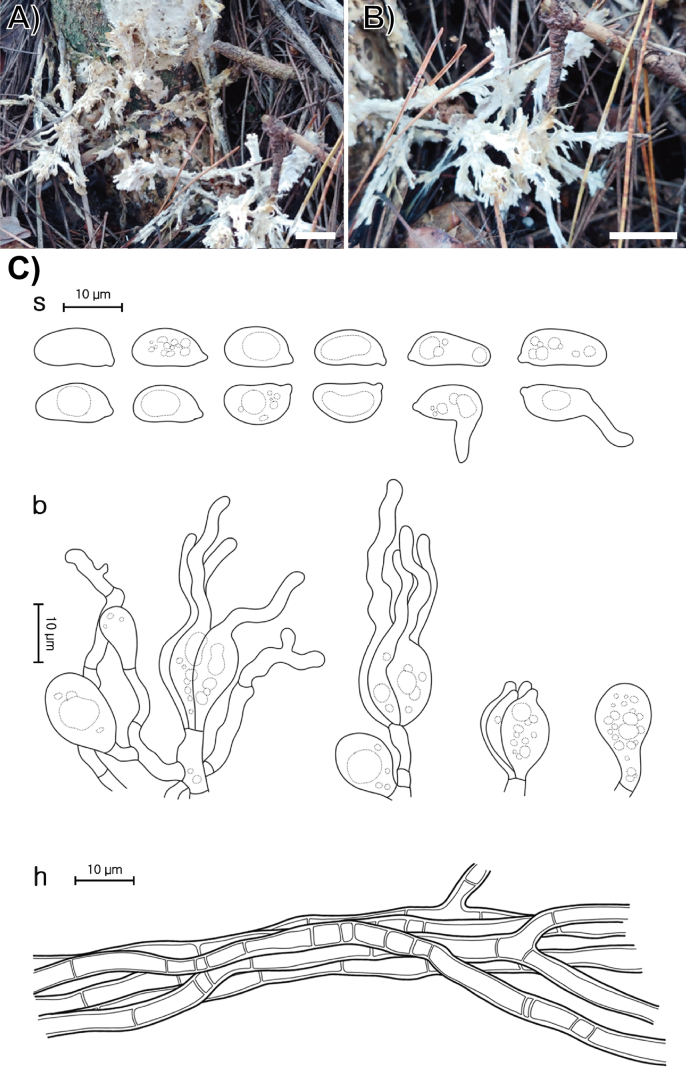
Morphological characters of *Sebacina crystallina* (SFC20200919-11, holotype). **A, B** basidiomata; **C** microscopic features, where ‘s’ refers to basidiospores, ‘b’ to basidia, basidioles, and dikaryophyses, and ‘h’ to hyphae. Scale bars: 1 cm (**A, B**).

**Figure 7.**
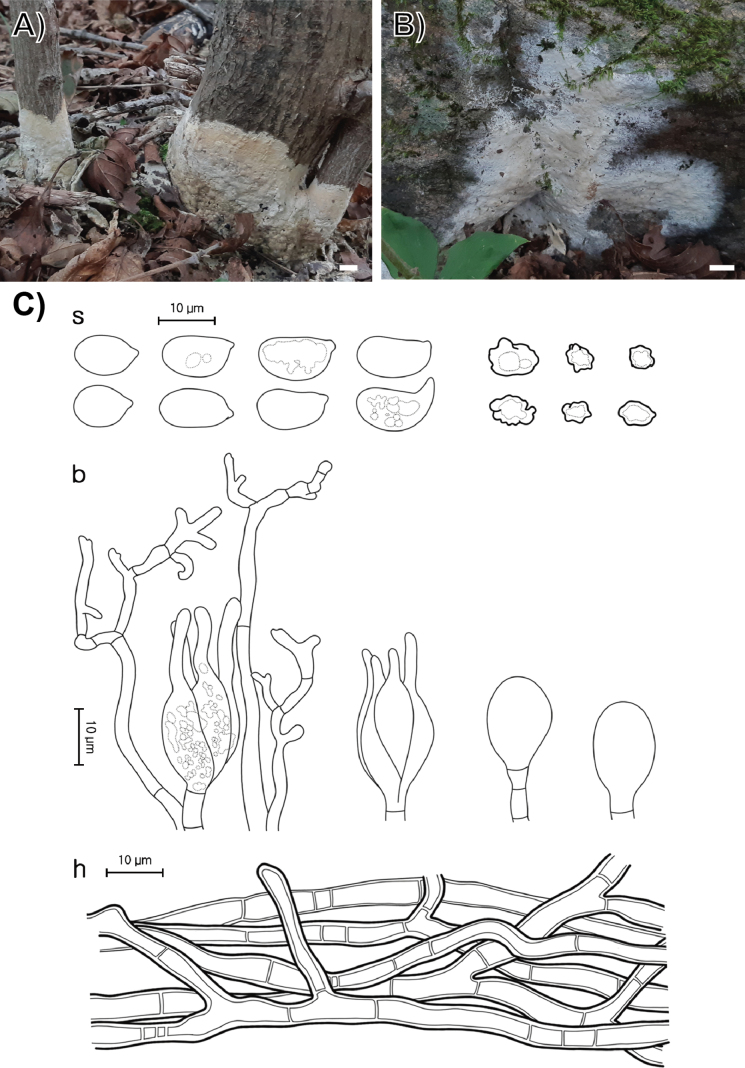
Morphological characters of *Sebacina incarnata*. **A** basidiomata (SFC20200821-21, holotype); **B** basidiomata (SFC20200821-26); **C** microscopic features, where ‘s’ refers to basidiospores and resting spores, ‘b’ to basidia, basidioles, and dikaryophyses and dikaryophyses, and ‘h’ to hyphae. Scale bars: 1 cm (**A, B**).

**Figure 8.**
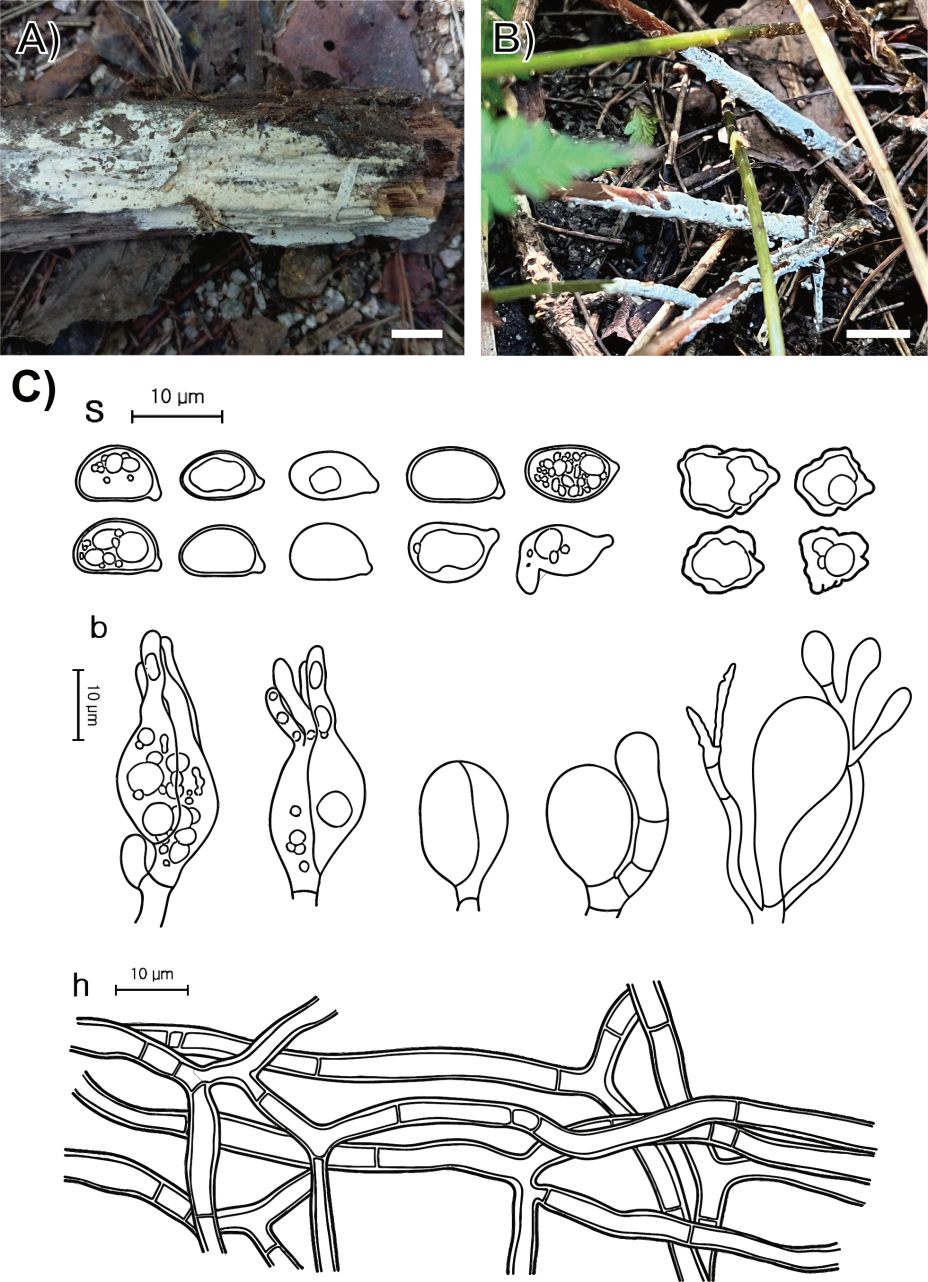
Morphological characters of *Sebacina orientalis*. **A** basidiomata (SFC20230908-13, holotype); **B** basidiomata (SFC20230908-04); **C** microscopic features, where ‘b’ refers to basidia, basidioles, and dikaryophyses, ‘s’ to basidiospores and resting spores, and ‘h’ to hyphae. Scale bars: 1 cm (**A, B**).

**Figure 9.**
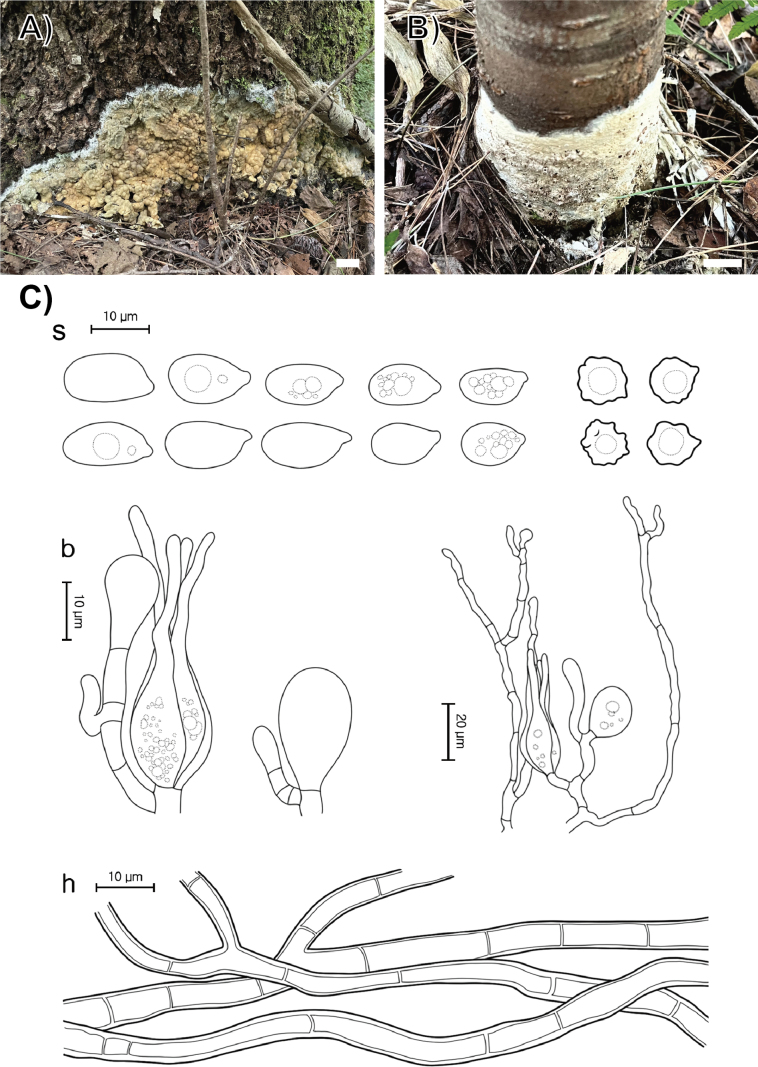
Morphological characters of *Sebacina aciculicola*. **A** basidiomata (SFC20230817-46, holotype); **B** basidiomata (SFC20230908-06); **C** microscopic features, where ‘s’ refers to basidiospores and resting spores, ‘b’ to basidia, basidioles, and dikaryophyses, and ‘h’ to hyphae. Scale bars: 1 cm (**A, B**).

**Table 1.** 

Species	Voucher	GenBank accession number			Country	Reference
		ITS	LSU	*rpb2*		
*G. rolleyi*	RJB 6889	–	AF291317	–	Canada	Weiß and Oberwinkler 2001
*Paulisebacina allantoidea*	RoKi 179	KF061266	AF291367	KF061301	Germany	Weiß and Oberwinkler 2001
*S. dimitica*	MW 525	–	AF291364	–	Germany	Weiß and Oberwinkler 2001
*S. incrustans*	MW 524	AF490395	AF291365	–	Germany	Weiß and Oberwinkler 2001

## Additional information

### Use of AI

AI was used to refine and clarify sentences.

### Adherence to national and international regulations

All the fungal strains used in this study have been legally obtained, respecting the Convention on Biological Diversity (Rio Convention).

### Author ORCIDs

Hannah Suh https://orcid.org/0009-0003-7526-1731

Chang Wan Seo https://orcid.org/0000-0002-5948-1836

Ki Hyeong Park https://orcid.org/0000-0002-8832-7643

Shinnam Yoo https://orcid.org/0000-0003-3686-3759

Dohye Kim https://orcid.org/0009-0007-0551-099X

Yoonhee Cho https://orcid.org/0000-0002-0743-0914

Young Woon Lim https://orcid.org/0000-0003-2864-3449

### Data availability

GenBank accessions for basidiomes: ITS PV399920–PV399957; LSU PV399883PV399919; *rpb2*PV417251–PV417276. GenBank accessions for root tip metabarcod ing data: ITS PV422624–PV422663; LSU PV422665–PV422704. Raw metabarcoding data used in this study are available at the NCBI Sequence Read Archive (https://www.ncbi.nlm.nih.gov/) under BioProject ID PRJNA894948.

## Supplementary materials

Supplementary material 1 Additional information Data type docx https://binary.pensoft.net/file/1548723This dataset is made available under the Open Database License (http://opendatacommons.org/licenses/odbl/1.0/). The Open Database License (ODbL) is a license agreement intended to allow users to freely share, modify, and use this Dataset while maintaining this same freedom for others, provided that the original source and author(s) are credited. Hannah Suh, Chang Wan Seo, Ki Hyeong Park, Shinnam Yoo, Dohye Kim, Yoonhee Cho, Young Woon Lim

Supplementary material 2 Alignment file of *Sebacinaceae* Data type fasta https://binary.pensoft.net/file/1548724This dataset is made available under the Open Database License (http://opendatacommons.org/licenses/odbl/1.0/). The Open Database License (ODbL) is a license agreement intended to allow users to freely share, modify, and use this Dataset while maintaining this same freedom for others, provided that the original source and author(s) are credited. Hannah Suh, Chang Wan Seo, Ki Hyeong Park, Shinnam Yoo, Dohye Kim, Yoonhee Cho, Young Woon Lim
