# Supplementary material for: ﻿Hidden diversity of crust-like Sebacinaceae (Sebacinales, Agaricomycetes) in Asia
Source: IMA Fungus. 2026 Jan 14;17:e168486. doi: 10.3897/imafungus.17.168486 (PMC12824589; doi:10.3897/imafungus.17.168486)
Supplement: Supplementary material 1 — Additional information [file imafungus-17-e168486-s001.docx]

Supporting Information for:

**Hidden diversity of crust-like Sebacinaceae (Sebacinales, Agaricomycetes) in Asia**

**Hannah Suh^1^,** **Chang Wan Seo ^1^, Ki Hyeong Park ^1,2^, Shinnam Yoo ^1^, Dohye Kim ^1^, Yoonhee Cho^1,3^, Young Woon Lim^1,*^**

^1^School of Biological Sciences and Institute of Biodiversity, Seoul National University, Seoul, Republic of Korea

^2^Forest Entomology and Pathology Division, National Institute of Forest Science, Seoul, Republic of Korea

^3^Department of Forest Biomaterials and Technology, Swedish University of Agricultural Sciences, Uppsala, Sweden

*Corresponding author Young Woon Lim, [ywlim@snu.ac.kr](mailto:ywlim@snu.ac.kr)

**Author Notes:**

*Corresponding author


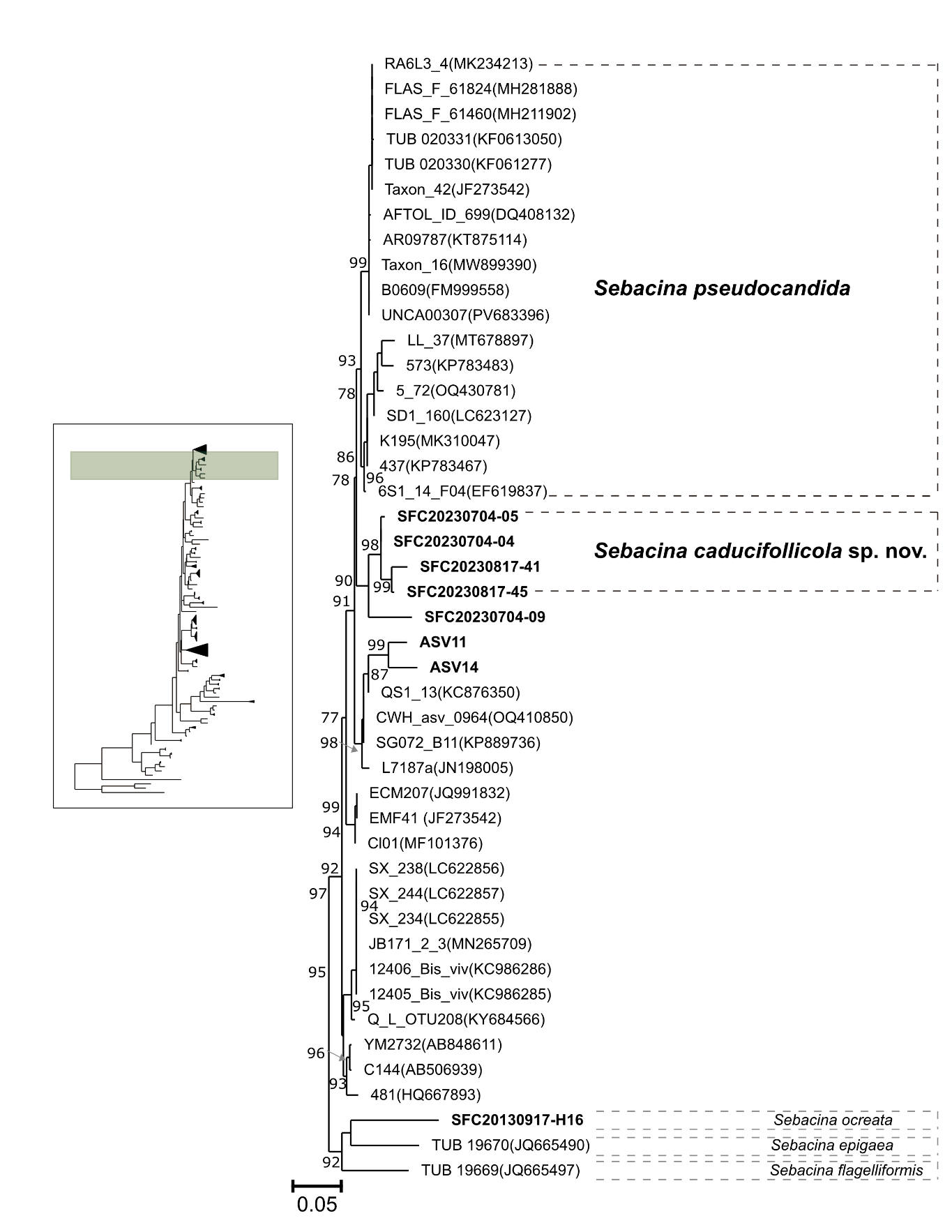


Figure S1. Phylogenetic tree inferred from Maximum Likelihood (ML) analysis based on ITS sequences of *Sebacina caducifollicola* sp. nov. and its close relatives, including sequences showing ≥94% similarity to *S. caducifollicola*. ML bootstrap support values above 70% are shown at the nodes. Sequences obtained from basidiomata and ASVs in this study are shown in bold. The names of formally described species are indicated by dashed boxes.


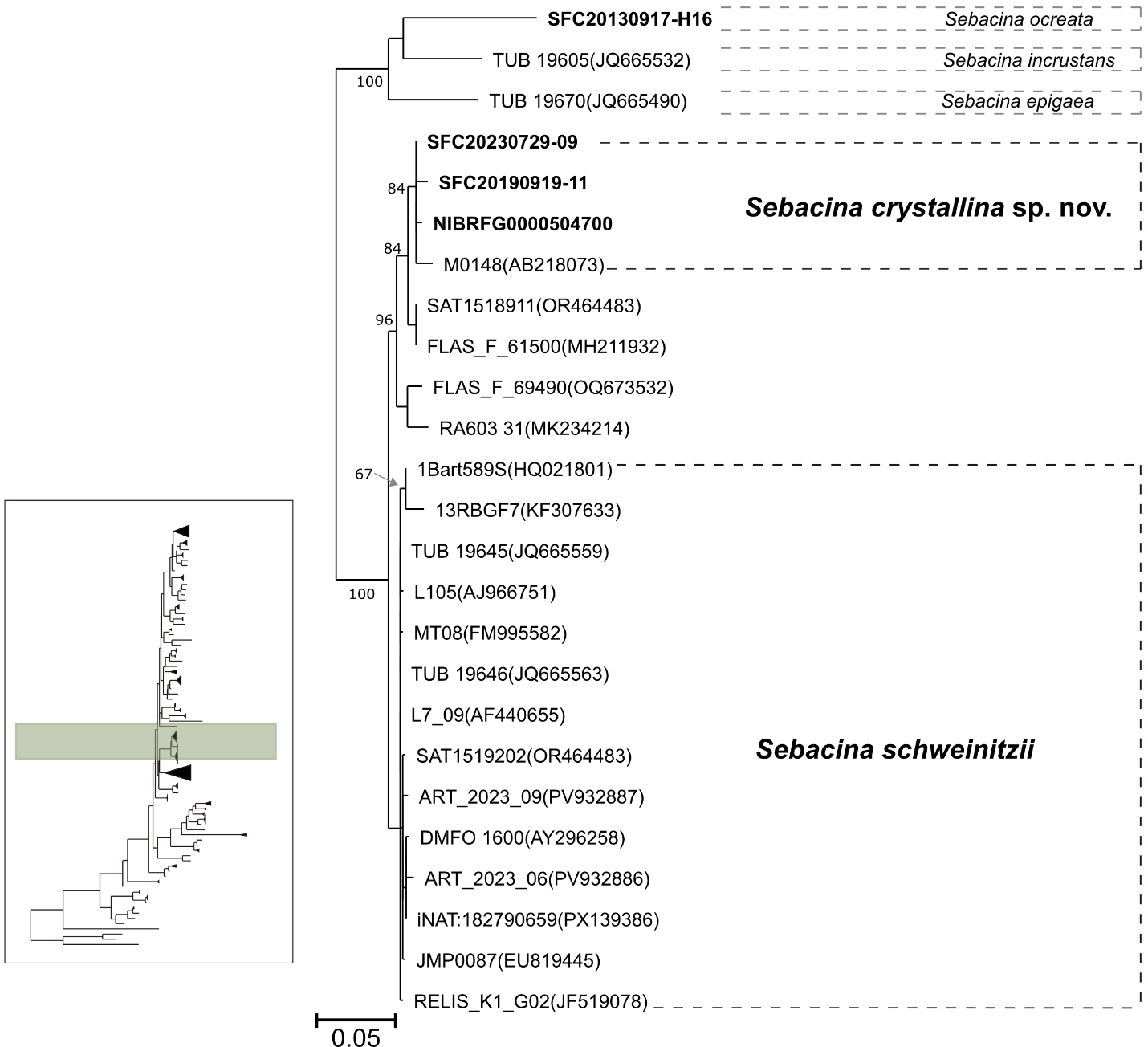


Figure S2. Phylogenetic tree inferred from Maximum Likelihood (ML) analysis based on ITS sequences of *Sebacina crystallina* sp. nov. and its close relatives, including sequences showing ≥94% similarity to *S. crystallina*. ML bootstrap support values above 70% are shown at the nodes. Sequences obtained from basidiomata and ASVs in this study are shown in bold. The names of formally described species are indicated by boxes.


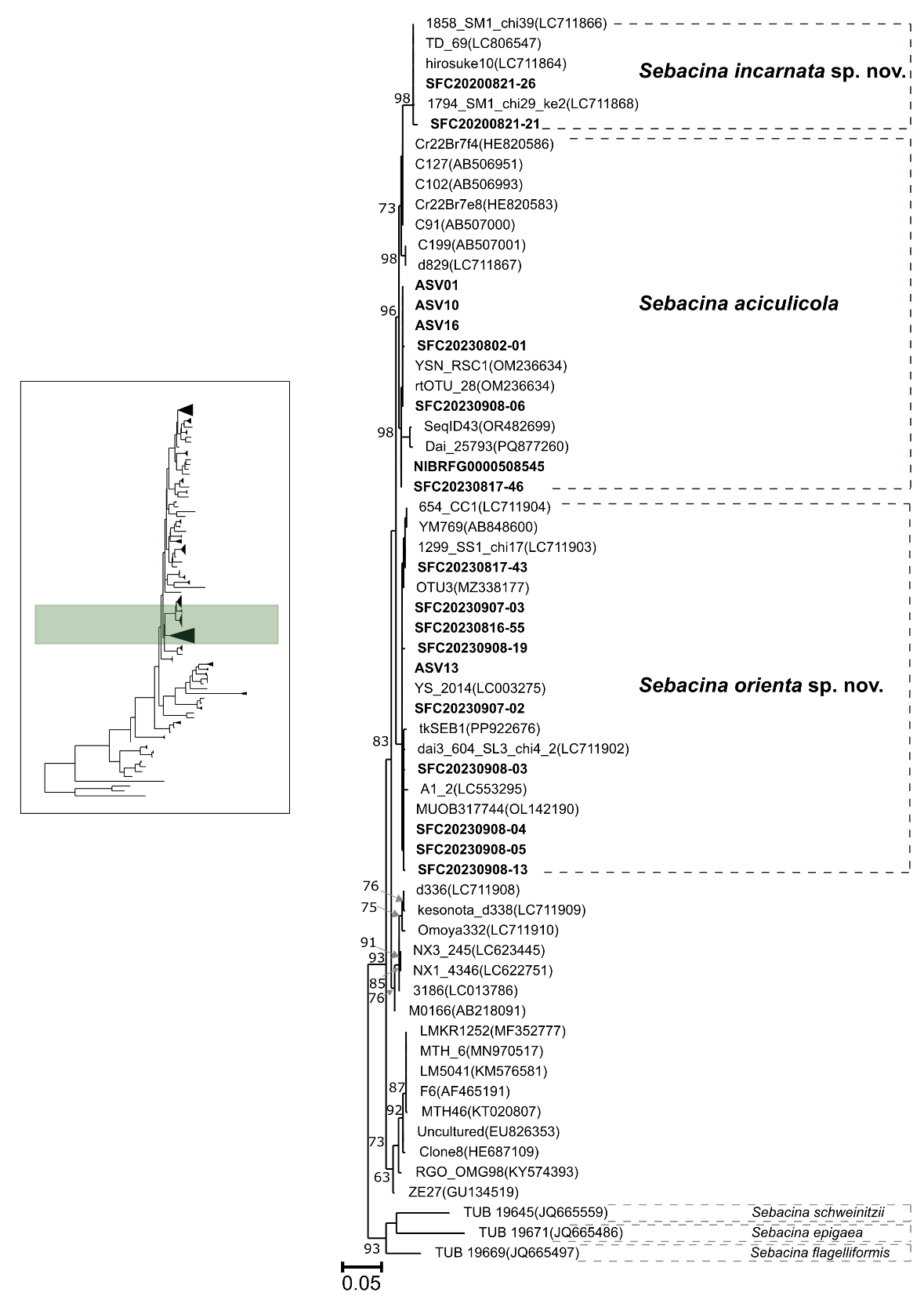


Figure S3. Phylogenetic tree inferred from Maximum Likelihood (ML) analysis based on ITS sequences of *Sebacina incarnata* sp. nov., *S. orienta* sp. nov., and their close relatives, including sequences showing ≥94% similarity to *S. incarnata* and *S. orienta*. ML bootstrap support values above 70% are shown at the nodes. Sequences obtained from basidiomata and ASVs in this study are shown in bold. The names of formally described species are indicated by dashed boxes.


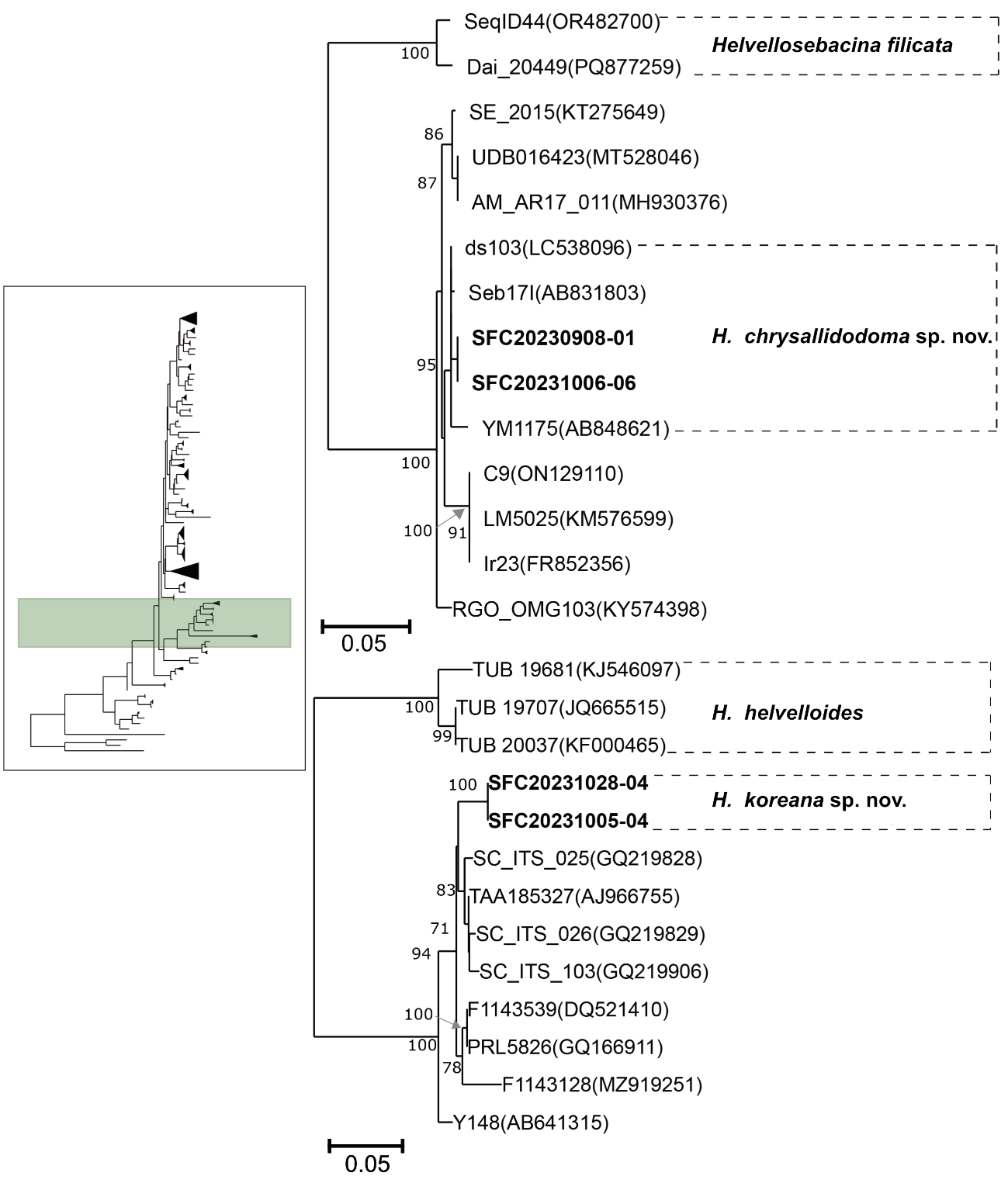


Figure S4. Phylogenetic tree inferred from Maximum Likelihood (ML) analysis based on ITS sequences of *Helvellosebacina chrysallidodoma* sp. nov., *H. koreana* sp. nov., and their close relatives, including sequences showing ≥94% similarity to *H. chrysallidodoma* and *H. koreana*. ML bootstrap support values above 70% are shown at the nodes. Sequences obtained from basidiomata and ASVs in this study are shown in bold. The names of formally described species are indicated by dashed boxes.
